# Defective lipid droplet biogenesis exacerbates oleic acid-induced cellular homeostasis disruption and ferroptosis in mouse cardiac endothelial cells

**DOI:** 10.1038/s41420-025-02669-5

**Published:** 2025-08-09

**Authors:** Yun-Ting Wang, Alexandra K. Moura, Rui Zuo, Zhengchao Wang, Kiana Roudbari, Jenny Z. Hu, Mi Wang, Pin-Lan Li, Yang Zhang, Xiang Li

**Affiliations:** 1 Department of Pharmacological and Pharmaceutical Sciences, College of Pharmacy, University of Houston, Houston, TX USA; 2 Provincial Key Laboratory for Developmental Biology and Neurosciences, College of Life Sciences, Fujian Normal University, Fuzhou, China; 3 Department of Gastroenterology and Hepatology, Tongji Hospital, Tongji Medical College, Huazhong University of Science and Technology, Wuhan, Hubei China; 4 Department of Pharmacology and Toxicology, Virginia Commonwealth University, School of Medicine, Richmond, VA USA

**Keywords:** Carotid artery disease, Fat metabolism, Cell death

## Abstract

Endothelial dysfunction is a hallmark of various metabolic disorders and plays a pivotal role in the progression of cardiovascular diseases, including coronary microvascular dysfunction and myocardial ischemia. Lipid droplets (LDs) have emerged as key regulators of fatty acid metabolism in endothelial cells (ECs), but their functional role in lipotoxicity-induced EC damage in the context of coronary microvascular dysfunction remains unclear. Here, we examined the contribution of LD biogenesis to oleic acid-induced lipotoxic effects in mouse cardiac ECs (MCECs). Our findings reveal that oleic acid markedly increases LD biogenesis in MCECs via a diacylglycerol O-acyltransferase 1 (DGAT1)-dependent pathway. This process is accompanied by substantial disruptions in cellular homeostasis, including elevated endoplasmic reticulum (ER) stress, impaired mitochondrial respiration, reduced ATP production, and heightened hypoxic responses. Furthermore, oleic acid-induced lipotoxicity is primarily mediated by ferroptosis−a form of lipid peroxide-dependent, caspase-independent cell death. Notably, pharmacological inhibition or genetic knockdown of DGAT1, which diminishes LD biogenesis, exacerbates oleic acid-induced cellular stress, mitochondrial dysfunction, and ferroptosis in MCECs. These results suggest that LD biogenesis plays a protective role in mitigating lipotoxicity, preserving mitochondrial function, and preventing lipid peroxide accumulation and ferroptosis, thereby safeguarding cardiac microvascular endothelial function in the context of metabolic disorders.

## Introduction

Metabolic disorders, such as obesity, hypercholesterolemia, and type 2 diabetes mellitus, are significant risk factors for exacerbating cardiovascular diseases, including coronary microvascular dysfunction and myocardial ischemia [1–3]. Endothelial dysfunction is an early onset of cardiovascular dysfunction associated with metabolic disorders, characterized by reduced nitric oxide bioavailability, increased oxidative stress, and an imbalance between vasodilatory and vasoconstrictive mediators [2, 4]. Lipids, particularly free fatty acids, play a critical role in the pathogenesis of endothelial dysfunction. Excessive free fatty acids can induce lipotoxicity, disrupt cellular homeostasis and cause endoplasmic reticulum (ER) stress and mitochondrial dysfunction in endothelial cells (ECs) [5, 6]. These lipotoxic effects lead to oxidative stress, inflammation, and cell death, thus impairing nitric oxide production, disrupting blood flow, increasing vascular permeability, and promoting a pro-inflammatory and pro-thrombotic environment. Collectively, these effects accelerate the progression of cardiovascular dysfunction in metabolic disorders [6, 7]. Despite these insights, the precise effects and regulatory mechanisms of lipids in coronary microvascular endothelial dysfunction remain incompletely understood.

Lipid droplets (LDs) are dynamic organelles that play a critical role in lipid storage and energy regulation in mammalian cells, including human and murine ECs, and influence a wide range of physiological and pathological processes [8, 9]. The abundance of LDs is determined by a dynamic balance between LD biogenesis and catabolism. LD biogenesis begins in the cytoplasmic leaflet of the ER, where neutral lipids such as triglycerides and cholesterol esters accumulate between the ER bilayer, forming a lens-like structure that buds off into the cytoplasm as a nascent LD surrounded by a phospholipid monolayer [10]. Proteins such as Diacylglycerol O-acyltransferase 1 (DGAT1) and DGAT2 are critical for triglyceride synthesis during this process, while perilipins (PLINs) and other LD-associated proteins regulate LD maturation, stability, and interactions with other organelles [11]. LD catabolism, on the other hand, begins with the degradation of LD membrane-coating proteins, particularly the Perilipin family member PLIN2, a substrate of chaperone-mediated autophagy. During chaperone-mediated autophagy, the KFERQ domain of PLIN2 is recognized by heat shock cognate protein 70 (HSC70), which delivers PLIN2 to the lysosomal lumen via lysosome-associated membrane protein 2A (LAMP2A) [12]. This process allows cytosolic lipases, such as adipose triglyceride lipase (ATGL), to access the LD core and initiate lipolysis. Alternatively, LDs can be degraded via macroautophagy, wherein autophagosomes sequester LDs, fuse with lysosomes, and catabolize LD cargos through lysosomal acid lipase [13].

Recent studies have revealed that abnormal LD abundance-whether insufficient or excessive-due to an imbalance in biogenesis and catabolism is linked to the metabolic state of the ECs and the progression of cardiovascular diseases [9, 14–16]. LDs provide cellular protection against lipotoxicity by buffering excess free fatty acids, cholesterols, and ceramides, storing them in acylated forms. Insufficient LD biogenesis, however, facilitates the accumulation of lipotoxic intermediates, which disrupt cellular homeostasis by causing ER stress and mitochondrial dysfunction, and eventually lead to cell death [17–19]. On the other hand, excessive LD accumulation has been linked to vascular inflammation and impaired nitric oxide production, potentially contributing to endothelial dysfunction and cardiovascular diseases [14, 16, 20, 21]. Our recent study demonstrates that coronary microvascular dysfunction is associated with increased lipid deposition in coronary arteriolar walls and upregulation of LD biogenesis-related genes [22]. However, the precise role of such LD accumulation in coronary microvascular endothelial dysfunction remains to be determined.

This study aimed to elucidate the role of LD biogenesis in mediating the effects of lipotoxicity on EC homeostasis and survival in the context of coronary microvascular dysfunction. Oleic acid, a monounsaturated omega-9 fatty acid abundant in animal and plant oils, is known to induce mitochondrial damage [23–25] and promote LD formation in ECs [8, 9]. Using cultured mouse cardiac endothelial cells (MCECs) as an oleic acid-induced lipotoxicity model, we identified DGAT1, but not DGAT2, as a key factor in LD biogenesis. By employing pharmacological and genetic inhibition of DGAT1, we evaluated the impact of impaired LD biogenesis on various lipotoxic effects of oleic acid, including heightened ER stress, mitochondrial dysfunction, and hypoxia. Ferroptosis is a regulated form of cell death driven by iron-dependent lipid peroxidation. Given the emerging recognition of ferroptosis as a significant contributor to EC death under lipid overload and oxidative stress [26, 27], we further investigated whether LD biogenesis defects exacerbate cell death triggered by oleic acid overload and whether this exacerbation is mediated by lipid peroxidation and ferroptosis. Our findings provide new insights into the therapeutic potential of targeting LD biogenesis to treat vascular complications, such as coronary microvascular dysfunction associated with metabolic disorders.

## Results

### Oleic acid induces LD biogenesis in MCECs

**Fig. 1:**
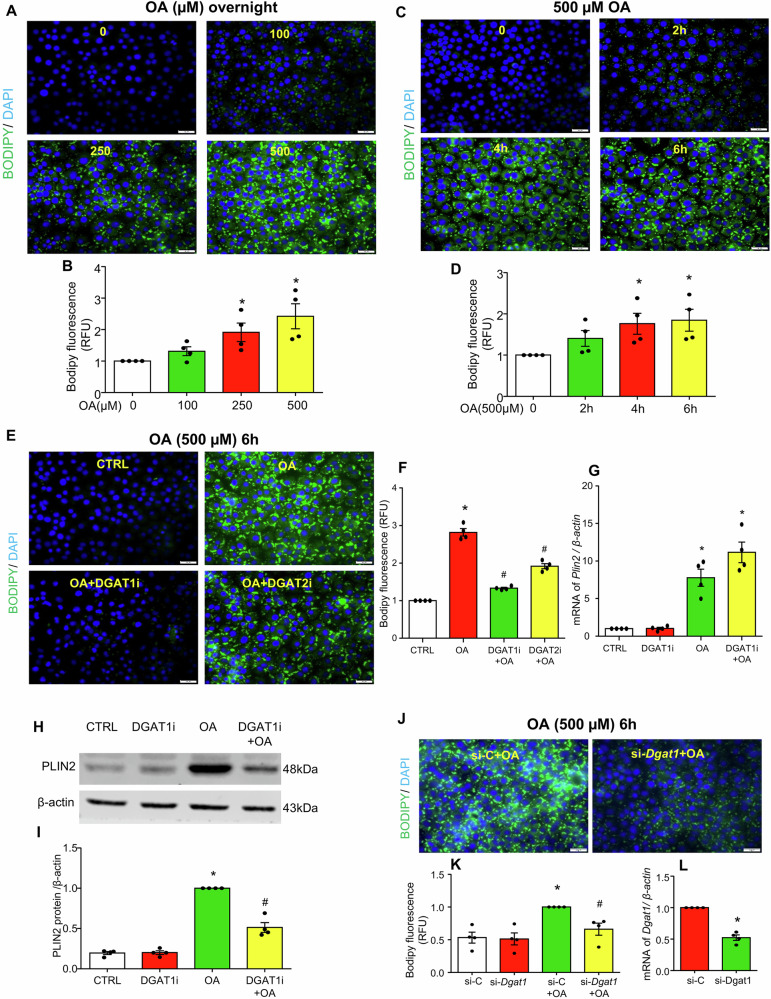
OA induces LD formation via DGAT1 in cultured MCECs. MCECs were seeded overnight, and upon reaching full confluence, were treated with the indicated dose of oleic acid (OA) for the specified times. **A**–**D** Representative immunofluorescence images of Bodipy staining and plate-read fluorescence intensity quantification showing that OA increased LD formation in a dose- and time-dependent manner. **E**–**I** MCECs were pretreated with or without 5 µM DGAT1 inhibitor A-922500 (DGAT1i) or 10 µM DGAT2 inhibitor PF-06424439 (DGAT2i) overnight, followed by treatment with 500 µM OA for 6 h. Representative Bodipy-stained images (**E**) and plate-read fluorescence intensity quantification (**F**) indicated that DGAT1 inhibition blocked OA-induced LD formation. **G** *Plin2* mRNA levels were measured, showing no significant effect of DGAT1 inhibition on transcription. **H**, **I** Representative immunoblots and quantification of PLIN2 protein levels demonstrate that DGAT1 inhibition significantly reduces OA-induced PLIN2 expression. Original uncropped WB bands for Fig. 1H were presented in Supplementary Fig. 3. **J**–**L** MCECs were transfected with 20 nM siRNA targeting *Dgat1* (si-*Dgat1*) or control siRNA (si-*Ctrl*) for 24 h, followed by 500 µM OA treatment for 6 h. Bodipy staining (**J**) and plate-reader fluorescence intensity quantification (**K**) show that *Dgat1* knockdown blocked OA-induced LD formation. **L** Dgat1 mRNA levels confirming successful knockdown. RFU, relative fluorescence unit. Images in **A**, **C**, **E** and J were acquired using a 40× objective and a standard 10× eyepiece (effective ×400 magnification). Scale bar =20 µm. Data in **B**, **D**, **F**, **G**, and **L** are shown as fold-changes relative to control (CTRL = 1), whereas **I** and **K** are normalized to the OA group (OA = 1). All datasets analyzed by the Kruskal–Wallis test with Dunn’s multiple-comparisons post hoc test (**B**, **D**, **F**–**H**, **K**) or by the Mann–Whitney test (**L**). *P < 0.05 vs. 0 (**B**, **D**), CTRL (**F**–**H**), or si-C (**K**, **L**); ^#^*P* < 0.05 vs. OA (**F**, **I**) or si-C + OA (**K**). *n* = 4.

Oleic acid is a monounsaturated fatty acid and a major component of membrane phospholipids that has been found to induce LD biogenesis in various ECs [9, 14, 21, 28–30]. To visualize oleic acid-induced LDs, Bodipy staining was performed in cultured MCECs. It was found that oleic acid dose- and time-dependently increased LD formation as evidenced by increased Bodipy fluorescence (Fig. 1A–D). DGAT1 inhibitor A-922500 (DGAT1i) effectively abolished oleic acid-induced LD formation, whereas DGAT2 inhibitor PF-06424439 (DGAT2i) only partially reduced LD formation (Fig. 1E, F). PLIN2 is a structural component of LDs in ECs [28]. DGAT1i did not attenuate oleic acid-induced transcriptional upregulation of *Plin2* (Fig. 1G), but significantly reduced PLIN2 protein expression (Fig. 1H, I). This uncoupling of transcript and protein levels suggests a compensatory increase in *Plin2* transcription in response to impaired lipid droplet biogenesis upon DGAT1 blockade. Consistently, *Dgat1* gene silencing also blocked oleic acid-induced increases in LD formation (Fig. 1J–L). These findings suggest that oleic acid increases LD biogenesis primarily through DGAT1 activity in MCECs.

### Effect of DGAT1 inhibition on lipogenesis, lipolysis, and fatty acid oxidation

**Fig. 2:**
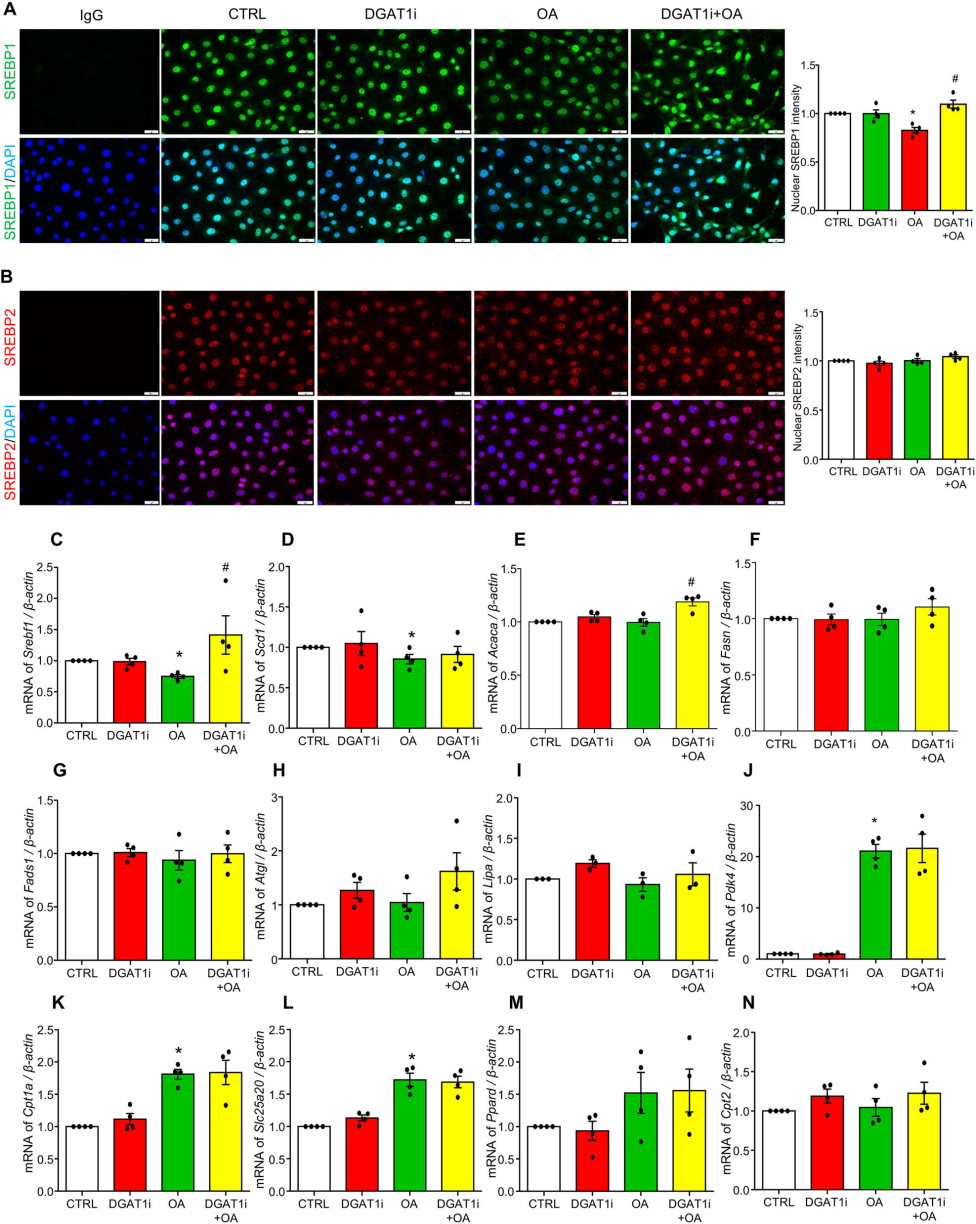
Effect of OA and DGAT1 inhibition on lipogenesis, lipolysis, and fatty acid oxidation (FAO). MCECs were seeded overnight, and upon reaching sub-confluence, were pre-treated with or without 5 µM DGAT1 inhibitor A-922500 (DGAT1i) for 30 min, followed by treatment with 250 µM OA for 6 h. **A**, **B** Representative immunofluorescence images of SREBP1 or 2, and their nuclear intensities summary. An isotype-matched IgG control (mouse or rabbit) was included as a negative control to confirm staining specificity. **C**–**G** Transcriptional level of key lipogenesis genes, including *Srebf1*, *Scd1, Acaca*, *Fasn* and *Fads1*. **H**, **I** Transcriptional level of lipolysis genes: *Atgl* and *Lipa*. **J**–**N** Transcriptional level of FAO genes, including *Pdk4*, *Cpt1α*, *Slc25a20, Pparβ/δ*, and *Cpt2*. Images in **A** and **B** acquired using a 40× objective and a standard 10× eyepiece (effective ×400 magnification). Scale bar =20 µm. Data are shown as fold-changes relative to control (CTRL = 1). All datasets were analyzed by the Kruskal–Wallis test with Dunn’s multiple-comparisons post hoc test. * vs CTRL, # vs OA, *P* < 0.05, *n* = 4.

Next, the role of DGAT1 in regulating lipogenesis, lipolysis, and fatty acid oxidation was evaluated in MCECs treated with oleic acid and DGAT1 inhibitor A-922500 (DGAT1i). Sterol regulatory element binding protein 1 (SREBP1), encoded by the sterol regulatory element binding transcription factor 1 (*Srebf1)* gene, is a key transcriptional regulator of fatty acid biosynthesis, whereas SREBP2, encoded by the *Srebf2* gene, primarily regulates cholesterol biosynthesis. As shown in Fig. 2A, immunofluorescence analysis showed that oleic acid decreased nuclear SREBP1 levels, whereas DGAT1 inhibition markedly increased nuclear SREBP1 compared with oleic acid alone. In contrast, neither oleic acid nor DGAT1i affected the nuclear expression of SREBP2 (Fig. 2B). Nuclear SREBP1 levels paralleled the changes in *Srebf1* mRNA expression (Fig. 2C). Notably, SREBP1 and SREBP2 are predominantly nuclear in our MCECs, likely because cells were maintained in low-glucose, low-serum medium. This nutrient deprivation depletes intracellular sterols and fatty acids, releases the SREBP cleavage–activating protein (SCAP) from insulin-induced gene (INSIG)-mediated ER retention, and triggers the proteolytic activation of SREBPs [31]. As a result, a large fraction of the cleaved, transcriptionally active SREBP1/2 accumulates in the nucleus under these baseline conditions. Oleic acid decreased mRNA levels of the *Srebf1* target gene stearoyl-CoA desaturase 1 (*Scd1*) (Fig. 2D), whereas transcripts of other *Srebf1*-regulated genes, acetyl-CoA carboxylase alpha (*Acaca*) (Fig. 2E), fatty acid synthase (*Fasn*) (Fig. 2F), and fatty acid desaturase 1 (*Fads1*) (Fig. 2G), were largely unaffected. Analysis of lipolysis-related genes, including adipose triglyceride lipase (*Atgl*) (Fig. 2H) and lysosomal acid lipase (*Lipa*) (Fig. 2I) further revealed no significant changes among all treatment groups. Furthermore, the expression of fatty acid oxidation-related genes was assessed, including pyruvate dehydrogenase kinase 4 (*Pdk4*), carnitine palmitoyltransferase 1a (*Cpt1a*), carnitine palmitoyltransferase 2 (*Cpt2*), solute carrier family 25 member 20 (*Slc25a20*), and peroxisome proliferator-activated receptor delta (*Ppard*). Oleic acid markedly upregulated the mRNA levels of *Pdk4* (Fig. 2J)*, Cpt1a* (Fig. 2K), *Slc25a20* (Fig. 2L), and showed an increasing trend for *Ppard* (Fig. 2M), while having no effect on *Cpt2* (Fig. 2N). Notably, DGAT1i did not further alter these effects, indicating that DGAT1 inhibition does not influence oleic acid-induced upregulation of the fatty acid oxidation pathway. Together, these results suggest that the impact of DGAT1 inhibition on LD formation is not due to impaired lipogenesis, enhanced lipolysis, or increased fatty acid oxidation but rather stems from a specific disruption of LD biogenesis.

### DGAT1 inhibition exacerbates oleic acid-induced ER stress in MCECs

**Fig. 3:**
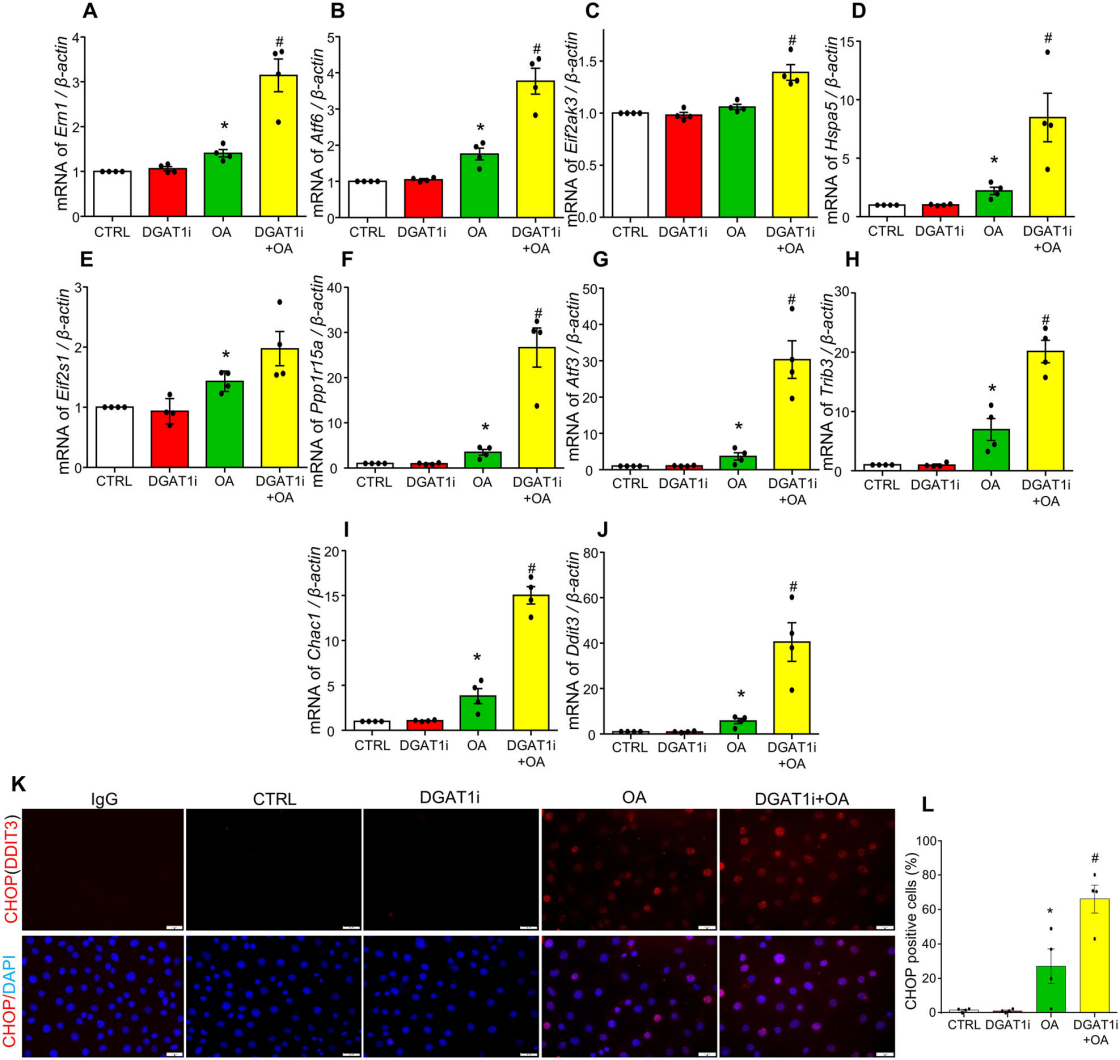
Inhibition of DGAT1 aggravates OA-induced ER-stress in cultured MCECs. MCECs were seeded overnight, and upon reaching sub-confluence, were pre-treated with or without 5 µM DGAT1 inhibitor A-922500 (DGAT1i) for 30 min, followed by treatment with 250 µM OA for 6 h. **A**–**J** Transcriptional level changes of ER-stress related genes, including *Ern1, Atf6, Eif2ak3, Bip, Eif2s1, Ppp1r15a, Atf3, Trib3, Chac1, and Ddit3*. **K**–**L** Representative immunofluorescence images of CHOP staining (**K**) and summary of positive cells percentage (**L**). An isotype-matched rabbit IgG control was included as a negative control to confirm staining specificity. Images in **K** acquired using a 40× objective and a standard 10× eyepiece (effective ×400 magnification). Scale bar = 20 µm. Data in **A**–**J** are shown as fold-changes relative to control (CTRL = 1). All datasets were analyzed by the Kruskal–Wallis test with Dunn’s multiple-comparisons post hoc test. * vs CTRL, # vs OA, *P* < 0.05, *n* = 4.

LD biogenesis takes place in the ER membrane and plays a critical role in mitigating the accumulation of free fatty acids and alleviating ER stress. To investigate whether DGAT1 inhibition by A-922500 (DGAT1i) enhances ER stress in MCECs, the gene expression of unfolded protein response signaling regulators were analyzed, including IRE1 (endoplasmic reticulum to nucleus signaling 1, gene symbol *Ern1*), activating transcription factor 6 (*Atf6*), and PERK (eukaryotic translation initiation factor 2 alpha kinase 3, gene symbol *Eif2ak3*). Furthermore, the downstream targets of these unfolded protein response pathways were examined, including ER chaperone GRP78/BIP (gene symbol *Hspa5*), eukaryotic translation initiation factor 2 subunit alpha (EIF2α, gene symbol *Eif2s1*), GADD34 (protein phosphatase 1 regulatory subunit 15 A, gene symbol *Ppp1r15a*), activating transcription factor 3 (*Atf3*), tribbles pseudokinase 3 (*Trib3*), ChaC glutathione-specific gamma-glutamylcyclotransferase 1 (*Chac1*), and CHOP (DNA damage inducible transcript 3, gene symbol *Ddit3*). Our results demonstrated that DGAT1 inhibition significantly intensified oleic acid-induced upregulation of *Ern1* (Fig. 3A), *Atf6* (Fig. 3B), and *Eif2ak3* (Fig. 3C). Consistent with these findings, as shown in Fig. 3D–J, DGAT1 inhibition enhanced oleic acid-induced increases in the expression of unfolded protein response downstream targets, including *Hspa5*, *Ppp1r15a*, *Atf3*, *Trib3*, *Chac1*, *Ddit3*, while also exhibiting an increasing trend for *Eif2s1*. Additionally, immunofluorescence studies confirmed that DGAT1 inhibition augmented oleic acid-induced increases in CHOP-positive cells (Fig. 3K–L), further indicating that DGAT1 inhibition intensifies ER stress in MCECs under lipotoxic conditions.

### DGAT1 inhibition exacerbates oleic acid-induced mitochondrial injury in MCECs

**Fig. 4:**
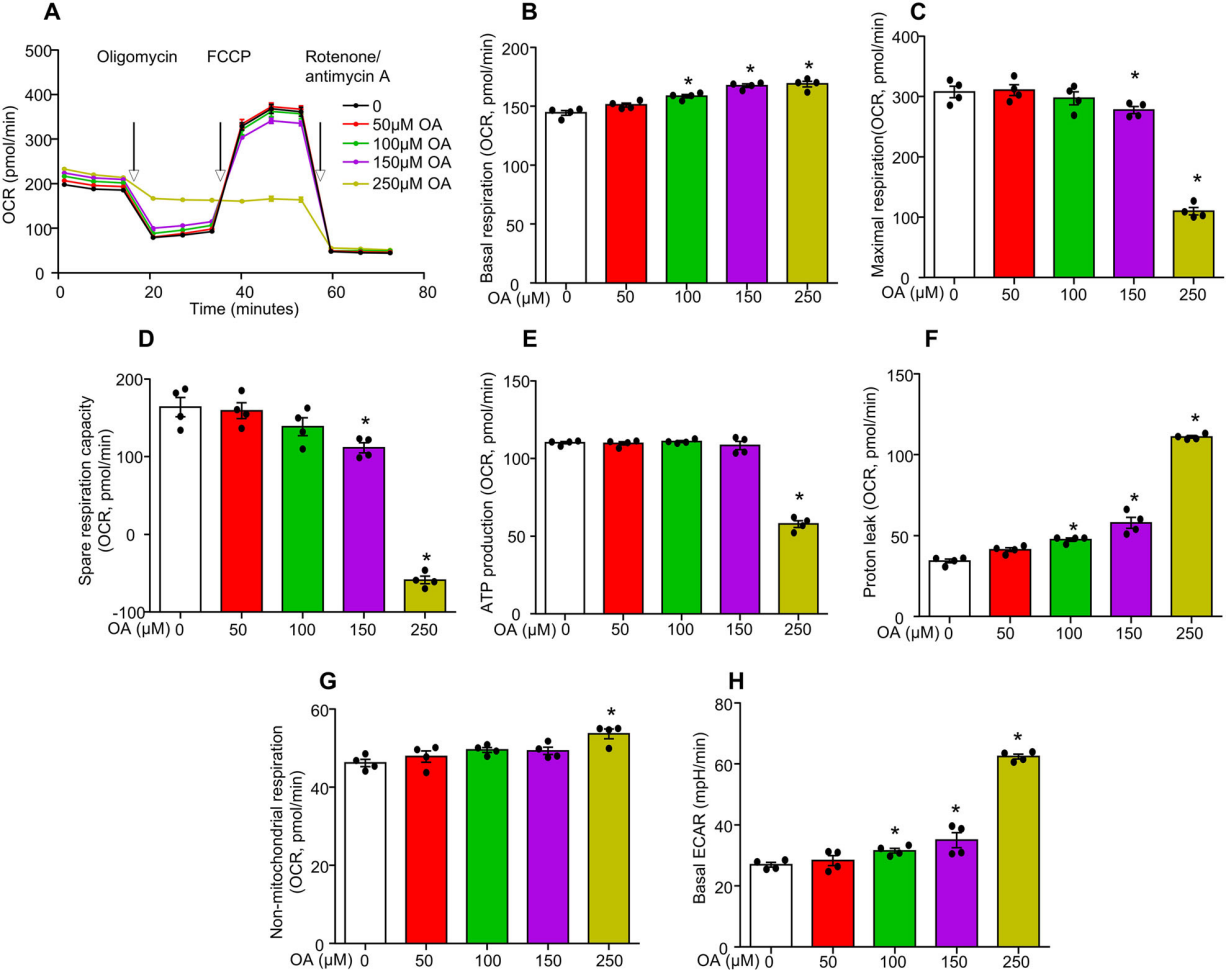
OA indued mitochondrial injury in cultured MCECs. MCECs were seeded overnight, and upon reaching sub-confluence, followed by treatment with different doses of OA for 6 h. **A**–**H** Effect of different doses of OA on mitochondrial functions, including basal respiration (**B**), maximal respiration (**C**), spare respiration capacity (**D**), ATP production (**E**), proton leak (**F**), non-mitochondrial respiration (**G**), and basal ECAR (**H**). All datasets were analyzed by the Kruskal–Wallis test with Dunn’s multiple-comparisons post hoc test. * vs 0, *P* < 0.05, *n* = 4. OCR, oxygen consumption rate, ECAR extracellular acidification rate.

The lipotoxic effects of oleic acid on MCECs in the absence of LDs were further assessed by evaluating mitochondrial function. A Seahorse assay was performed to measure the oxygen consumption rate (OCR), a critical indicator of mitochondrial activity. As shown in Fig. 4A, the oxygen consumption was analyzed in MCECs treated with various concentrations of oleic acid under basal conditions and in response to the ATPase inhibitor oligomycin, the protonophore FCCP, and the respiration chain inhibitors rotenone/antimycin A. The summarized data demonstrated that oleic acid treatment increased basal respiration as shown by increased oxygen consumption in a dose-dependent manner (Fig. 4B). Meanwhile, oleic acid significantly decreased maximal respiration (Fig. 4C), spare respiratory capacity (Fig. 4D), and ATP production (Fig. 4E). Notably, reductions in maximal respiration and spare respiration capacity were observed starting at 150 µM oleic acid, while significant ATP production impairment occurred only at 250 µM. These findings suggest that at lower concentrations (less than 150 µM), cells have mild mitochondrial damage but preserve mitochondrial function and ATP production. In contrast, higher concentrations (250 µM) of oleic acid induce a decompensated state characterized by severe mitochondrial dysfunction and compromised ATP production. Additionally, oleic acid dose-dependently increased proton leak across the mitochondrial inner membrane (Fig. 4F), a sign of mitochondrial damage. Lipotoxic effects on mitochondria were further associated with elevated non-mitochondrial respiration (Fig. 4G) and a rise in the basal extracellular acidification rate (ECAR) (Fig. 4H), indicating a metabolic shift toward anaerobic glycolysis under conditions of substantial mitochondrial damage.

**Fig. 5:**
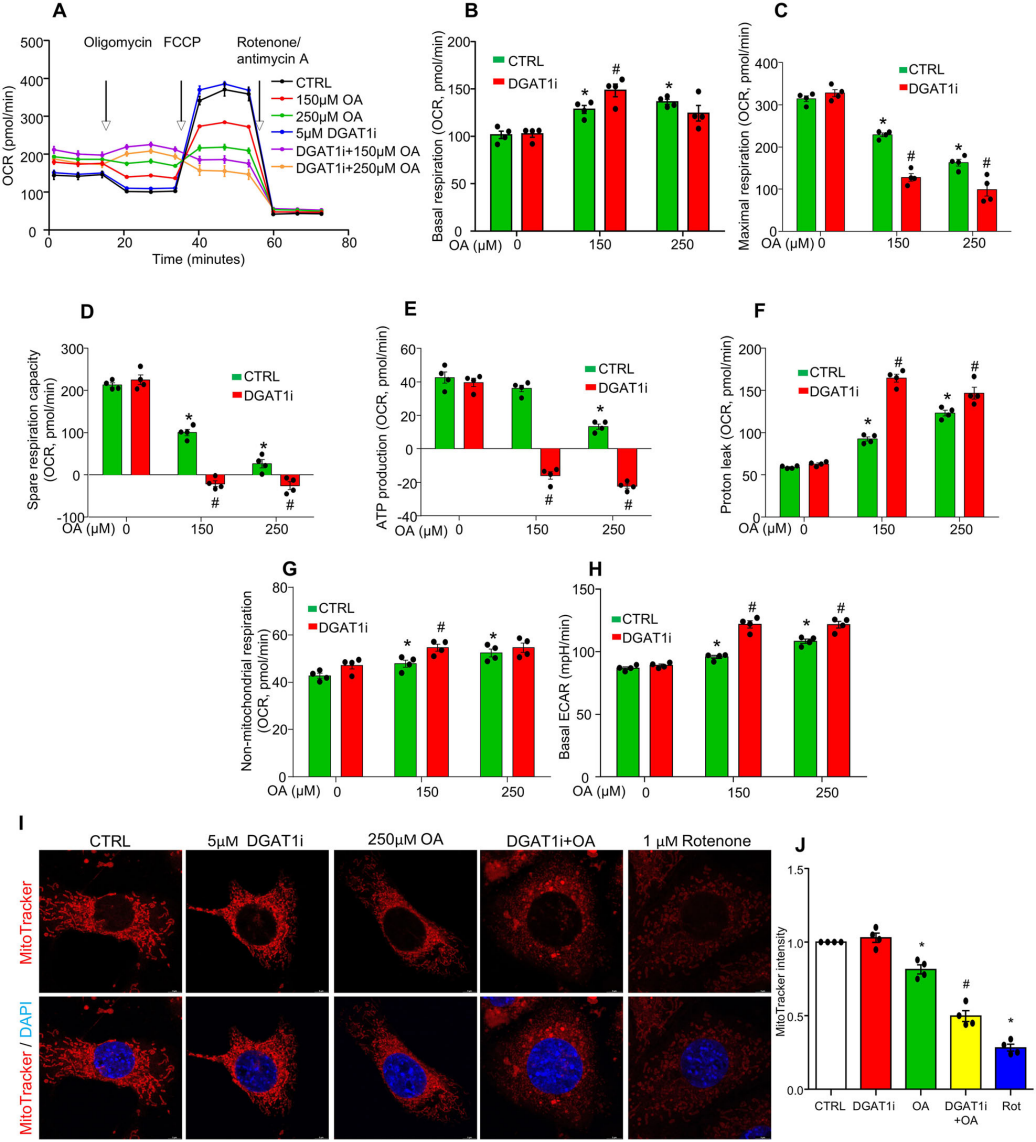
Inhibition of DGAT1 exacerbates OA-induced mitochondrial injury in cultured MCECs. MCECs were seeded overnight, and upon reaching sub-confluence, were pre-treated with or without 5 µM DGAT1 inhibitor A-922500 (DGAT1i) for 30 min, followed by treatment with indicated doses of OA for 6 h. **A–H** Effect of OA and DGAT1i on mitochondrial functions, including basal respiration (**B**), maximal respiration (**C**), spare respiration capacity (**D**), ATP production (**E**), proton leak (**F**), non-mitochondrial respiration (**G**), and basal ECAR (**H**). **I**, **J** Representative confocal images of MitoTracker-labeled mitochondria (**I**). Quantification of mean mitochondrial fluorescence intensity (**J**). 1 µM rotenone 6 h was included as a positive control for mitochondrial disruption. Data in **J** are shown as fold-changes relative to control (CTRL = 1). Images in **I** were acquired using a 100× oil-immersion objective and a standard 10× eyepiece, with 2× digital zoom (effective ×2000 magnification). Scale bar= 5 µm. All datasets were analyzed by the Kruskal–Wallis test with Dunn’s multiple-comparisons post hoc test. *P < 0.05 vs. CTRL without OA (0 µM), ^#^*P* < 0.05 vs. CTRL treated with the same concentration of OA. *n* = 4. OCR, oxygen consumption rate; ECAR, extracellular acidification rate.

Next, the role of defective LD biogenesis in exacerbating the lipotoxic effects of oleic acid on mitochondrial function was investigated by measuring oxygen consumption in MCECs treated with the DGAT1 inhibitor A-922500 (DGAT1i). DGAT1 inhibition significantly worsened oleic acid-induced mitochondrial dysfunction (Fig. 5A). Specifically, at a lower dose of 150 μM oleic acid, DGAT1 inhibition enhanced basal respiration, further decreased maximal respiration and spare respiration capacity, and led to ATP production failure compared to oleic acid treatment alone, where ATP production remained preserved (Fig. 5B–E). Furthermore, DGAT1 inhibition intensified the oleic acid-induced proton leak (Fig. 5F), increased non-mitochondrial respiration (Fig. 5G), and augmented ECAR levels (Fig. 5H). MitoTracker Deep Red staining showed that OA reduced mitochondrial fluorescence and disrupted network integrity; DGAT1 inhibition further exacerbated these defects, inducing pronounced fragmentation (Fig. 5I–J), underscoring that DGAT1 blockade worsens lipotoxic mitochondrial dysfunction in MCECs. Together, these results indicate that DGAT1 inhibition significantly exacerbates oleic acid-induced mitochondrial dysfunction and structural disruption, leading to ATP production failure and a pronounced shift to anaerobic glycolysis.

### DGAT1 inhibition enhances oleic acid-induced cellular hypoxia in MCECs

**Fig. 6:**
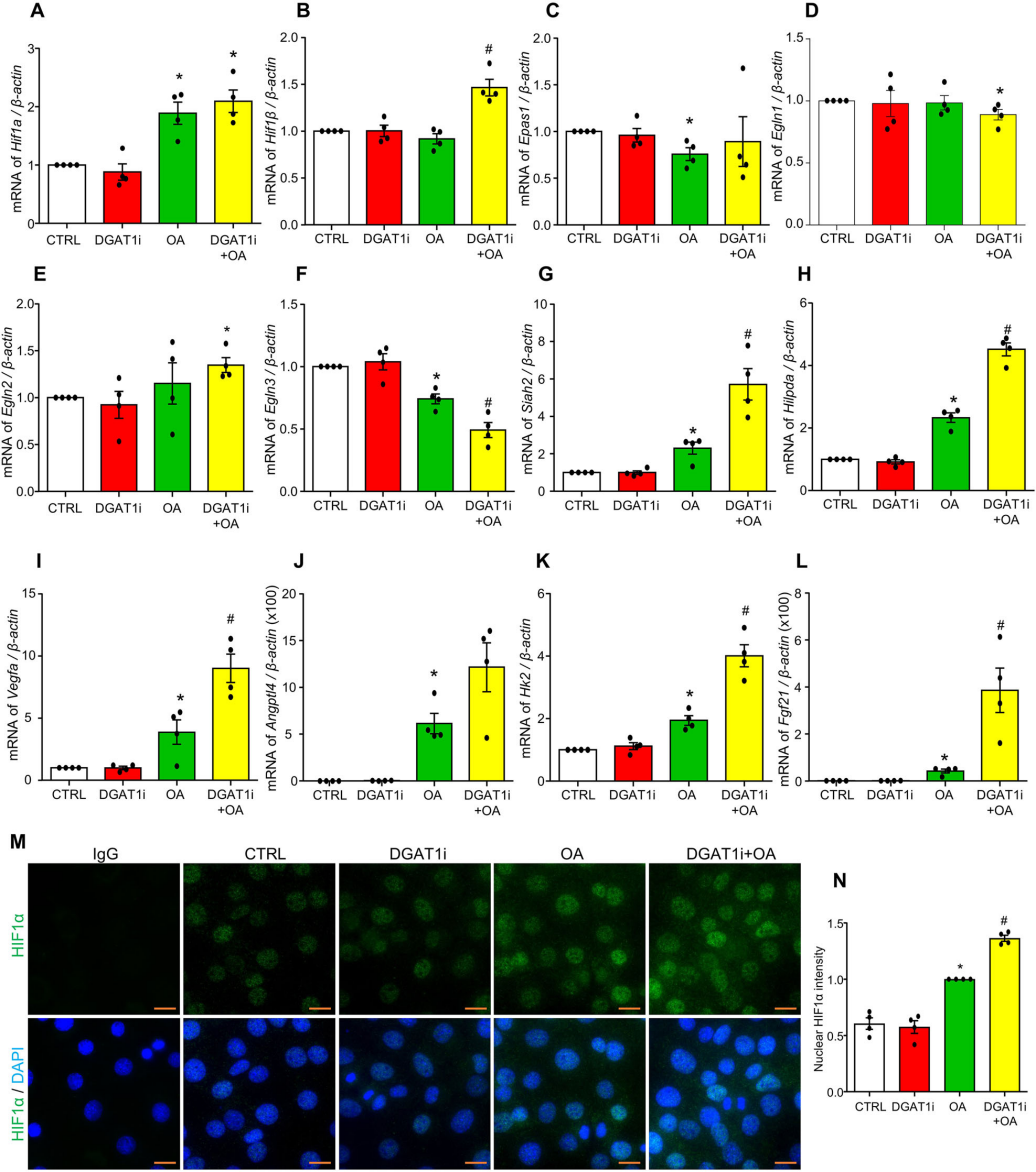
Inhibition of DGAT1 enhances OA-induced hypoxia in cultured MCECs. MCECs were seeded overnight, and upon reaching sub-confluence, were pre-treated with or without 5 µM DGAT1 inhibitor A-922500 (DGAT1i) for 30 min, followed by treatment with 250 µM OA for 6 h. **A**–**L** Transcriptional level changes of hypoxia-related genes, including *Hif1a, Hif1β, Epas1(Hif2), Egln1, Egln2, Egln3, Siah2, Hilpda, Vegfa, Angptl4, Hk2, and Fgf21*. **M**, **N** Representative immunofluorescence images of HIF1a, and its nuclear intensities summary. An isotype-matched rabbit IgG control was included as a negative control to confirm staining specificity. Data in **A**–**L** are shown as fold-changes relative to control, while data in **N** are shown as fold-changes relative to OA. Images in **M** acquired using a 63× oil-immersion objective and a standard 10× eyepiece (effective ×630 magnification). Scale bar = 10 µm. All datasets were analyzed by the Kruskal–Wallis test with Dunn’s multiple-comparisons post hoc test. * vs CTRL, # vs OA, *P* < 0.05, *n* = 4.

Oleic acid decreases oxygen consumption, which may lead to cellular hypoxia and activation of hypoxia-inducible factors (HIFs), key regulators of glycolysis that facilitate cellular adaptation to low oxygen conditions and maintain homeostasis under hypoxia [32]. To investigate the effects of DGAT1 inhibition by A-922500 (DGAT1i) on oleic acid-induced cellular hypoxia, the mRNA levels of HIF pathway-related genes were examined. These included HIF-1α (*Hif1α*), HIF-1β (*Hif1β*), HIF-2 (encoded by *Epas1* gene), prolyl hydroxylase domain-containing enzyme PHD2 (encoded by *Egln1* gene), PHD1 (encoded by *Egln2* gene), PHD3 (encoded by *Egln3* gene), as well as Siah E3 Ubiquitin Protein Ligase 2 (*Siah2*), which mediates the ubiquitination and degradation of PHD1 and PHD3. As shown in Fig. 6A–G, oleic acid significantly increased the mRNA levels of *Hif1α* and *Siah2*, and downregulated *Epas1* and *Egln3*, with no change in *Hif1β* or *Egln1*; *Egln2* was unaffected by OA alone but was significantly increased by OA + DGAT1i. These findings suggest that oleic acid, under normoxic conditions yet with impaired mitochondrial respiration, triggers a “pseudohypoxic response” [33], which was associated with activation of HIF-1α and SIAH2, and suppression of PHD3. Notably, co-treatment of oleic acid with DGAT1i resulted in significantly higher expression levels of *Hif1β* and *Siah2*, but lower *Egln3*, compared to treatment with oleic acid alone (Fig. 6A–G). This impact of DGAT1 inhibition on the hypoxia pathway was confirmed by a significant augmentation of HIF1 targeted genes, including Hypoxia Inducible Lipid Droplet Associated (*Hilpda*), Vascular Endothelial Growth Factor A (*Vegfa)*, Angiopoietin like 4 (*Angptl4)*, and glycolysis-related gene Hexokinase 2 (*Hk2*) (Fig. 6H–K). Additionally, fibroblast growth factor 21 (*Fgf21*), a glycolysis-associated gene not directly regulated by HIF-1α, was also upregulated by oleic acid and further increased by DGAT1 inhibition (Fig. 6L). In addition to the transcriptional data, we confirmed OA increased nuclear HIF-1α protein levels, and DGAT1 inhibition further enhanced this effect (Fig. 6M, N).

### DGAT1 inhibition exacerbates oleic acid-induced ferroptosis in MCECs

**Fig. 7:**
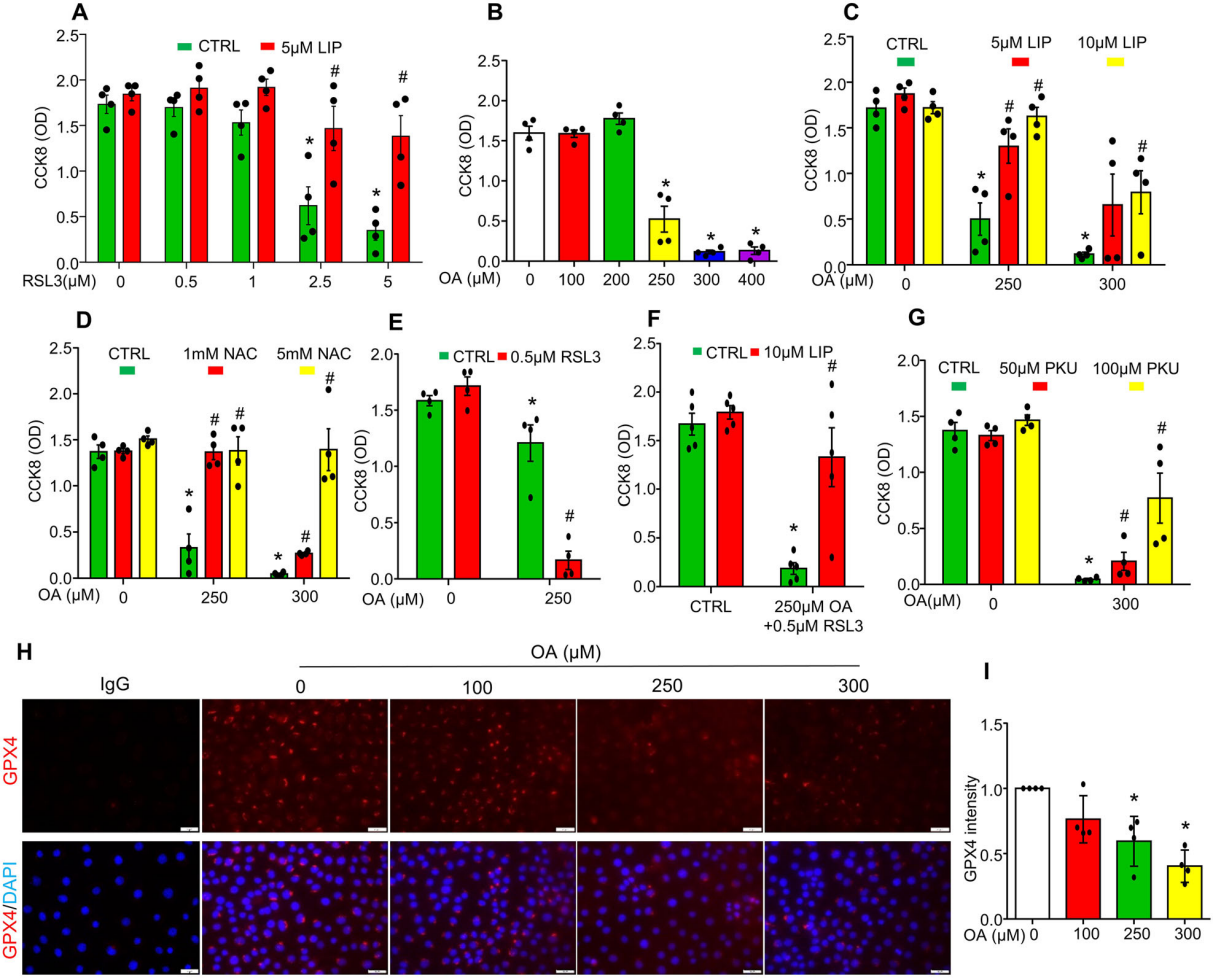
OA induces ferroptosis via inhibiting GPX4 in cultured MCECs. MCECs were seeded overnight, and upon reaching 70% confluence, were pre-treated with indicated inhibitors or activators for 30 min, followed by treatment with indicated doses OA for specified times. **A** Ferroptosis inhibitor liproxstatin (LIP) blocked GPX4 inhibitor RSL3-induced cell death after overnight treatment. **B** OA dose-dependently induced cell death in MCECs after overnight treatment. Effect of LIP (**C**) and ROS inhibitor (N-Acetyl-L-cysteine (NAC)) (**D**) on OA-induced cell death. **E** RSL3 sensitized OA-induced cell death. **F** LIP mitigated cell death induced by the combination of OA and RSL-3. **G** GPX4 activator PKUMDL-LC-101-D04 (PKU) rescued cells from OA-induced cell death. **H**, **I** Representative immunofluorescence images and intensity quantification showing overnight OA treatment decreased GPX4 expression. An isotype-matched rabbit IgG control was included as a negative control to confirm staining specificity. Images in **H** acquired using a 40× objective and a standard 10× eyepiece (effective ×400 magnification). Scale bar = 20 µm. Data in **I** are shown as fold-changes relative to control (CTRL = 1). All datasets were analyzed by the Kruskal–Wallis test with Dunn’s multiple-comparisons post hoc test. *P < 0.05 vs. corresponding CTRL lacking RSL3 (**A**), lacking OA (**B**, **I**), or lacking OA and additional treatments (**C**–**G**); ^#^*P* < 0.05 vs. CTRL treated with the same concentration of RSL3 (**A**), the same concentration of OA (**C**, **D**, **E**, **G**), or OA + RSL3 (**F**). *n* = 4. OD, optical density.

Ferroptosis is a type of programmed cell death characterized by the accumulation of lipid peroxides and iron dependency. GPX4 is the key regulator of ferroptosis occurrence, mainly by inhibiting the formation of lipid peroxides [27]. As shown in Fig. 7A, GPX4 inhibitor (1S,3R)-RSL3 (RSL3) induced cell death in MCECs, evidenced by a dose-dependent decrease in cell viability. This effect was significantly reversed by ferroptosis inhibitor liproxstatin-1 (LIP) (Fig. 7A), suggesting that GPX4 inhibition is sufficient to trigger ferroptosis in MCECs. Oleic acid also caused dose-dependent cell death in MCECs starting at 250 μM (Fig. 7B). Notably, this oleic acid-induced cell death was significantly reversed by liproxstatin-1 (Fig. 7C) and the antioxidant N-acetyl-L-cysteine (NAC) (Fig. 7D). In contrast, inhibitors of other programmed cell death pathways (apoptosis, pyroptosis, autophagic cell death), including the pan-caspase inhibitor Z-VAD-FMK [34, 35], caspase-3 inhibitor Z-DEVD-FMK [36], caspase-1 inhibitor AC-YVAD-CMK [37], lysosomal inhibitors chloroquine (CQ) [38] and Bafilomycin A1 (BAF) [39], and the autophagosome inhibitor spautin-1 (SP-1) [40], failed to protect MCECs from oleic acid-induced cell death (Supplementary Fig. 1). Thus, these results suggest that oleic acid primarily induces lipid peroxide accumulation and ferroptotic cell death in MCECs but not apoptosis, pyroptosis, or autophagic cell death. Additionally, RSL3 at a non-cytotoxic dose (0.5 µM) sensitized MCECs to oleic acid-induced ferroptosis, an effect that was abrogated by liproxstatin-1 (Fig. 7E, F). Conversely, stimulation of GPX4 by PKUMDL-LC-101-D04 (PKU), a novel allosteric GPX4 activator, significantly rescued cells from oleic acid-induced cell death (Fig. 7G). Immunofluorescence staining revealed that oleic acid treatment suppressed the protein expression of the endogenous ferroptosis inhibitors GPX4 in a dose-dependent manner in MCECs (Fig. 7H, I). Together, these findings suggest that oleic acid-induced ferroptosis is associated with a decreased availability of the endogenous ferroptosis inhibitor.

**Fig. 8:**
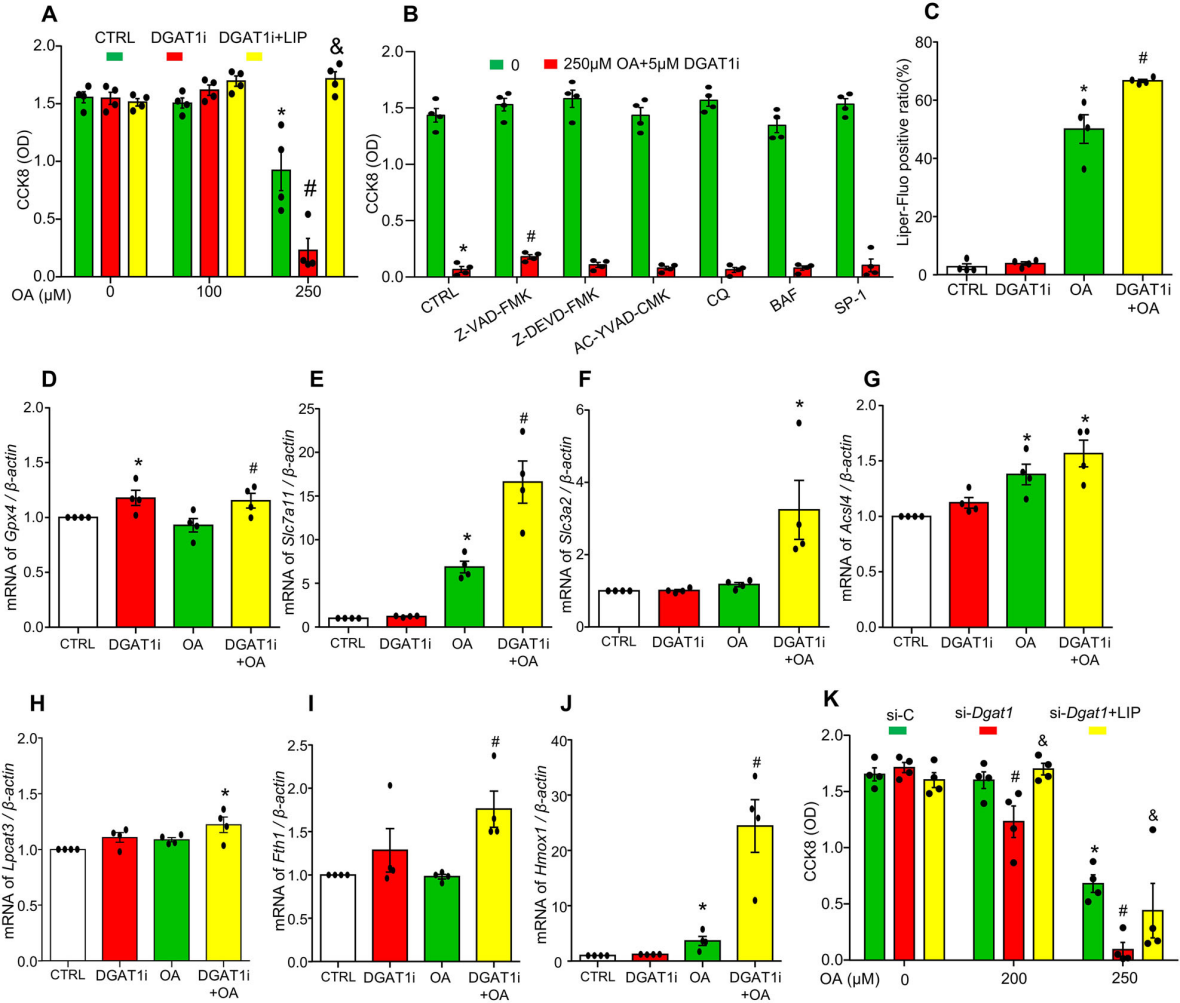
DGAT1-mediated LD biogenesis protects against OA-induced ferroptosis in cultured MCECs. MCECs were seeded overnight, and upon reaching 70% confluence, were pre-treated with indicated inhibitors for 30 min, followed by treatment with indicated doses OA for specified times. **A** Inhibition of DGAT1 exacerbated OA-induced ferroptosis. **B** Pan-caspase inhibitor (50 µM Z-VAD-FMK), caspase-3 inhibitor (20 µM Z-DEVD-FMK), caspase-1 inhibitor (30 µg/ml AC-YVAD-CMK), lysosome inhibitors chloroquine (5 µM CQ) and bafilomycin (10 nM BAF), and autophagy inhibitor spautin-1 (10 µM SP-1) had minimal or no effect on OA and DGAT1i-induced cell death. **C** Flow cytometric analysis revealed DGAT1 inhibition enhanced OA-induced lipid peroxidation measured by Liper-Fluo, a key marker for ferroptosis. **D**–**J** Transcriptional changes in ferroptosis-related genes, including *Gpx4, Slc7a11, Slc3a2, Acsl4, Lpcat3, Fth1 and Hmox1*. **K** Dgat1 gene knockdown exacerbated OA-induced ferroptosis. Data in **D**–**J** are shown as fold-changes relative to control (CTRL = 1). All datasets were analyzed by the Kruskal–Wallis test with Dunn’s multiple-comparisons post hoc test. In **A**, **K**, *P < 0.05 vs. corresponding CTRL or si-C lacking OA and additional treatments; ^#^*P* < 0.05 vs. CTRL or si-C treated with the same concentration of OA; & *P* < 0.05 vs. DGAT1i or si-*Dgat1* with same concentration of OA. In **B**, *P < 0.05 vs. corresponding CTRL lacking OA and DGAT1i; ^#^*P* < 0.05 vs. CTRL treated OA + DGAT1i. In **C**–**J**, *P <0.05  vs CTRL, #*P* *<0.05*  vs OA. *n* = 4. OD, optical density.

To investigate whether defective LD biogenesis promotes oleic acid-induced ferroptosis, the cytotoxic effects of the DGAT1 inhibitor A-922500 (DGAT1i) on MCECs was examined. As shown in Fig. 8A, DGAT1i alone had no significant effect on cell viability but markedly intensified oleic acid (250 µM)-induced cell death, which was completely reversed by the ferroptosis inhibitor liproxstatin-1. In contrast, Fig. 8B demonstrates that inhibitors of non-ferroptotic cell death pathways, including Z-VAD-FMK, Z-DEVD-FMK, AC-YVAD-CMK, chloroquine (CQ), Bafilomycin A1 (BAF), and spautin-1 (SP-1), had minimal or no protective effects against cell death induced by oleic acid combined with DGAT1i. Further, Fig. 8C shows that DGAT1 inhibition significantly enhanced oleic acid-induced lipid peroxide production. These findings suggest that in the absence of LD biogenesis, oleic acid-induced cell death is more pronounced with enhanced lipid peroxide accumulation and occurs primarily in the form of ferroptosis. To gain mechanistic insight into oleic acid-induced ferroptosis under conditions of defective LD biogenesis, the mRNA levels of ferroptosis pathway-related genes were examined, including *Gpx4*, system Xc- antiporter components (*Slc7a11 and Slc3a2*), acyl-CoA synthetase long-chain family member 4 (*Acsl4*), lysophosphatidylcholine acyltransferase 3 (*Lpcat3*), ferritin heavy chain 1 (*Fth1*), and heme oxygenase 1 (*Hmox1*). As shown in Fig. 8D–J, oleic acid treatment alone significantly upregulated *Slc7a11, Acsl4*, and *Hmox1* expression but had no effect on *Gpx4*, *Slc3a2*, *Lpcat3*, or *Fth1*. However, co-treatment with oleic acid and DGAT1i resulted in more pronounced increases in *Gpx4, Slc7a11*, *Slc3a2*, *Fth1*, and *Hmox1* expression compared to oleic acid alone. Finally, the role of DGAT1 in oleic acid-induced ferroptosis was confirmed using *Dgat1* gene silencing in MCECs. As shown in Fig. 8K, *Dgat1* knockdown exacerbated oleic acid-induced cell death, which was rescued by liproxstatin-1. Together, these results suggest that in the absence of LD biogenesis, oleic acid induces more severe ferroptosis, characterized by the intensified increases in lipid peroxide accumulation and upregulation of ferroptosis-related genes (*Gpx4, Slc7a11*, *Slc3a2*, *Fth1, Hmox1*) signaling pathways.

To extend these findings to human endothelial cells, we performed parallel experiments in HUVECs (Supplementary Fig. 2). As shown in Supplementary Fig. 2A, B, DGAT1 inhibition completely blocked OA-induced lipid droplet formation in HUVECs. In dose–response assays (Supplementary Fig. 2C), 250 μM OA caused significant cell death, mirroring the effects seen in MCECs. Importantly, DGAT1i further exacerbated OA-induced cell death in HUVECs, and this heightened cell death was rescued by liproxstatin-1 (Supplementary Fig. 2D). As in MCECs, Supplementary Fig. 2E shows that inhibitors of non-ferroptotic cell death pathways, including Z-VAD-FMK, Z-DEVD-FMK, AC-YVAD-CMK, CQ, BAF, and SP-1, had no protective effects against cell death induced by oleic acid combined with DGAT1i in HUVECs. These human-cell data reinforce our conclusion that, in the absence of DGAT1-dependent LD biogenesis, OA overload drives severe ferroptosis characterized by excessive lipid peroxidation and up-regulation of ferroptosis-related genes.

## Discussion

The goal of this study was to investigate the mechanisms and role of LD biogenesis in regulating oleic acid-induced lipotoxicity in MCECs. Our findings provide mechanistic insights regarding the biogenesis of LDs through a DGAT1-dependent pathway. Pharmacological inhibition or genetic knockdown of DGAT1 abolishes LD biogenesis, resulting in exacerbated lipotoxic effects characterized by increased ER stress, mitochondrial damage, and cellular hypoxia. These intensified lipotoxic effects ultimately promote the accumulation of lipid peroxides, leading to EC death through ferroptosis.

Recent studies have shown that LDs are the major intracellular organelles for lipid storage in human and murine ECs, particularly under conditions of lipid overload [8, 9]. LD accumulation is determined by the dynamic balance between LD biogenesis and catabolism. LD biogenesis begins in the ER, where neutral lipids like triglycerides, accumulate in the ER bilayer to form a lens-like structure, which buds off into the cytoplasm as a nascent LD surrounded by a phospholipid monolayer. In ECs, both DGAT1 and DGAT2 contribute to LD biogenesis, where PLIN2 serves as a primary LD-associated coating protein that promotes LD maturation and stability [9]. However, whether defects in LD biogenesis are linked to changes in lipogenesis or lipid catabolism in ECs remains unclear. In the present study, our data demonstrate that oleic acid-induced LD biogenesis in MCECs primarily depends on DGAT1, with DGAT2 playing only a minor role. Furthermore, our results show that the reduction in LD accumulation caused by DGAT1 inhibition is not associated with the downregulation of genes in lipogenesis or upregulation of genes in fatty acid oxidation and lipolysis in MCECs. To our knowledge, this study is the first to establish the critical role of DGAT1 in LD accumulation by specifically regulating biogenesis rather than catabolism in MCECs.

Lipotoxicity occurs when excessive accumulation of lipids, particularly free fatty acids, disrupts cellular homeostasis, resulting in dysfunction and damage. In the ER, lipid overload impairs protein folding and disrupts calcium homeostasis, triggering ER stress and activating the unfolded protein response [41]. This stress response aims to restore ER function, but sustained ER stress can lead to inflammation, oxidative stress, and cell death, linking lipotoxicity to metabolic syndrome and cardiovascular diseases [42]. Free fatty acids, such as palmitic acid and oleic acid, have been identified as potent inducers of ER stress in various mammalian cells [9, 43, 44]. Notably, ER stress has also been shown to trigger LD biogenesis under lipotoxic conditions to help maintain cellular lipid homeostasis [5]. Increased LD formation is often accompanied by elevated ER stress during lipid overload and is thought to mitigate lipotoxicity by sequestering excessive lipids [9]. ER stress is commonly assessed by monitoring the changes in unfolded protein response pathways. A recent study reported that palmitate-induced unfolded protein response in human umbilical vein ECs was exacerbated by DGAT1 inhibition but not by DGAT2 inhibition [45]. Similarly, in this study, we demonstrate that in MCECs, DGAT1 inhibition intensifies oleic acid-induced ER stress. This is characterized by heightened activation of key unfolded protein response regulators, including IRE1 (*Ern1*), ATF6 (*Atf6*), and PERK (*Eif2ak3*), and their downstream targets, such as BIP (*Hspa5*), EIF2 (*Eif2s1*), GADD34 (*Ppp1r15a*), ATF3 (*Atf3*), TRIB3 (*Trib3*), CHAC1 (*Chac1*), and CHOP (*Ddit3*). These findings support the view that DGAT1 is the key enzyme for triglyceride synthesis, facilitating LD biogenesis to sequester excess free fatty acids in MCECs. This highlights the critical importance of LD biogenesis in alleviating ER stress under lipotoxic conditions.

Mitochondria are highly sensitive to lipid overload, as excessive free fatty acid accumulation in mitochondrial membranes can disrupt mitochondrial function [46]. Previous studies have reported that oleic acid or its derivative, 12-hydroxy oleic acid, impairs mitochondrial function by reducing ATP production in human umbilical vein ECs [23, 24] and liver sinusoidal ECs [25]. However, oleic acid does not significantly impact mitochondrial function in mouse lung endothelial cells [9]. In this study, we demonstrate that oleic acid induces mitochondrial dysfunction in MCECs at higher concentrations (250 μM), as evidenced by impaired maximal respiration and reduced ATP production. These findings suggest that lipid overload from oleic acid overwhelms the sequestration capacity of LDs, leading to lipotoxicity and a decompensated state that compromises ATP production. Proton leak occurs when protons (H⁺), pumped into the intermembrane space by the electron transport chain (ETC), bypass ATP synthase and re-enter the mitochondrial matrix. Instead of contributing to ATP synthesis, these protons combine with oxygen to form water [47]. This process decreases oxidative phosphorylation efficiency, increases oxygen consumption, and dissipates energy as heat rather than generating ATP. Excessive proton leaks disrupt the mitochondrial membrane potential and reduce ATP production efficiency, contributing to mitochondrial dysfunction. Our data show that oleic acid significantly increases proton leaks in MCECs. While the precise mechanisms remain unclear, free fatty acids are known to act as proton carriers, shuttling protons across the mitochondrial membrane back into the matrix, thereby uncoupling the proton gradient from ATP synthesis [48]. Proteins such as uncoupling protein 2 (UCP2) in non-adipocytes may also mediate controlled proton leak [49]. These mechanisms likely contribute to oleic acid-induced proton leak and mitochondrial damage and warrant further investigation. Additionally, our findings indicate that oleic acid triggers a metabolic shift toward anaerobic glycolysis, characterized by elevated non-mitochondrial respiration and an increased extracellular acidification rate. This metabolic shift may represent an adaptive response to compensate for reduced ATP production caused by lipotoxicity-induced mitochondrial damage. Furthermore, we demonstrate that DGAT1 inhibition exacerbates oleic acid-induced mitochondrial damage, including further increased proton leak, failure of ATP production via the mitochondrial respiratory chain, and a more pronounced shift toward anaerobic glycolysis. These findings highlight the protective role of LD biogenesis in mitigating oleic acid-induced mitochondrial damage, preserving mitochondrial function, and reducing reliance on less efficient glycolytic pathways. In the absence of DGAT1 activity and LD biogenesis, cells shift toward glycolysis and non-mitochondrial respiration as a compensatory response to severe mitochondrial dysfunction.

Accumulating evidence indicates that LD biogenesis is closely linked to cellular adaptation under hypoxia, a condition characterized by low oxygen availability. Hypoxia-inducible factors (HIFs) play a central role in this adaptation by driving the expression of genes that regulate lipid metabolism, promoting LD formation to sequester excess free fatty acids and maintain lipid homeostasis, thereby protecting against lipotoxicity [50–52]. Additionally, HIFs enhance compensatory mechanisms such as angiogenesis and glycolysis, facilitating cellular survival during oxygen deprivation [32, 53, 54]. In cancer cells, LD accumulation is a hallmark of hypoxia, with HIF-1α acting as a master regulator to sustain cellular homeostasis and survival [32, 50, 55]. One mechanism linking hypoxia to LDs involves HIF-1α-mediated upregulation of HILPDA, a known inhibitor of adipose triglyceride lipase (ATGL), the key enzyme for intracellular lipolysis. This downregulation of LD catabolism enables cells to preserve stored lipids under hypoxic stress [56, 57]. HIF-1α and the structural subunit HIF-1β form the HIF-1 heterodimeric complex, thus becoming activated HIF-1α [58]. In the present study, we demonstrate that oleic acid significantly increased basal oxygen consumption (Fig. 5B) and activated the HIF-1α signaling pathway (Fig. 6), suggesting that oleic acid triggers hypoxic effects in MCECs. Notably, oleic acid upregulated *Siah2* expression while downregulating *Egln3* (PHD3), consistent with previous findings that SIAH2 mediates the ubiquitination and degradation of PHD3, thereby stabilizing and enhancing HIF-1α signaling [59, 60]. However, further investigation is warranted to elucidate the role of the SIAH2-PHD3-HIF-1α axis in oleic acid-induced hypoxic effects in MCECs. Among the HIF-1α target genes, *Hilpda* was significantly upregulated by oleic acid, potentially suppressing ATGL activity and linking hypoxia to reduced LD catabolism. Additionally, oleic acid increases the expression of genes associated with angiogenesis (*Vegfa, Angptl4*) and glycolysis (*Hk2*, *Fgf21*) [32, 53, 54]. These findings suggest that oleic acid-induced pseudohypoxic response activates HIF-1 signaling in MCECs to promote adaptive mechanisms such as LD accumulation, angiogenesis, and glycolysis, which helps mitigate lipotoxicity and mitochondrial damage. Furthermore, our study reveals that DGAT1 inhibition exacerbates oleic acid-induced pseudohypoxia, amplifying these adaptive pathways in MCECs. Notably, DGAT1 inhibition further enhances HIF-1α nuclear expression and HIF-1β gene expression, suggesting that DGAT1 inhibition may activate the HIF-1 signaling pathway through increased HIF-1α/β dimer formation. Collectively, these results confirm that LD biogenesis serves as a vital protective mechanism against lipotoxicity-induced hypoxia in MCECs, aiding in the maintenance of cellular homeostasis under lipid overload conditions.

The present study further elucidates the mechanism by which oleic acid-induced lipotoxicity leads to cell death. Ferroptosis, a type of programmed cell death, is characterized by the accumulation of lipid peroxides and dependence on iron [61]. While previous studies have shown that oleic acid induces ferroptosis in various mammalian cells or tissues [62, 63], its ferroptotic effects on ECs remain incompletely understood. In the present study, we demonstrate that oleic acid induces ferroptotic cell death in MCECs at concentrations starting from 250 μM. This cell death is significantly reversed by ferroptosis inhibitor liproxstatin-1 and the antioxidant N-acetyl-L-cysteine (NAC) but is unaffected by inhibitors of other programmed cell death pathways, such as apoptosis, pyroptosis, and autophagic cell death. At higher concentrations (300 μM), oleic acid-induced cell death is only partially attenuated by liproxstatin-1 but is completely blocked by antioxidant NAC. Notably, oleic acid has been reported to induce necrotic cell death in liver sinusoidal ECs [25], suggesting that at elevated levels, oleic acid may trigger ROS-dependent necroptotic cell death in MCECs. Nonetheless, our findings are the first to demonstrate that oleic acid-induced lipotoxicity in ECs primarily leads to cell death in the form of ferroptosis. Mechanistically, ferroptosis is regulated by endogenous inhibitor GPX4, a key regulator of ferroptosis, mainly by preventing the formation of lipid peroxides [61]. The levels of this inhibitor critically influence cell’s susceptibility to ferroptotic death, underscoring their role in both physiological and pathological contexts. Our data reveal that oleic acid significantly decreases GPX4 protein levels (Fig. 7), suggesting that its ferroptotic effects are associated with reduced availability of this inhibitor. Interestingly, oleic acid does not alter the mRNA expression of GPX4 (Fig. 8), indicating that their downregulation occurs through an autophagy-lysosome-independent but proteasome-dependent post-translational mechanism [64, 65].

Recent studies highlight that LD biogenesis induced by oleic acid exerts protective effects against ferroptosis by sequestering free fatty acids, thereby limiting lipid peroxidation [66–69]. Specifically, polyunsaturated fatty acids, which are highly susceptible to lipid peroxidation, are esterified by acyl-CoA synthetase long-chain family member 4 (ACSL4) and incorporated into membrane phospholipids by lysophosphatidylcholine acyltransferase 3 (LPCAT3) [70]. LDs act as antioxidant organelles by storing polyunsaturated fatty acids in triglycerides, thereby reducing membrane lipid peroxidation, and preventing ferroptosis [70]. Consistent with this, we demonstrate that DGAT1 inhibition exacerbates oleic acid-induced lipid peroxidation and ferroptosis in MCECs. This exacerbation is associated with increased upregulation of ferroptosis-related genes (*Gpx4, Slc7a11*, *Slc3a2*, *Fth1, Hmox1*). The increased expression of anti-ferroptotic factors (*Gpx4, Slc7a11*, *Slc3a2*, *Fth1*) in response to LD biogenesis defects may represent an adaptive mechanisms to counteract heightened lipid peroxidation. The role of HMOX1 in EC ferroptosis remains controversial [71]. On one hand, HMOX1 activation has been reported to mediate the protective effect of pigment epithelium-derived factor (PEDF) on ferroptosis in human cardiac microvascular ECs under hypoxia reoxygenation [72]. On the other hand, HMOX1 has been implicated in promoting EC ferroptosis in retinal neovascularization and diabetic atherosclerosis through increased iron content, ROS, and lipid peroxidation [73, 74]. These findings support a dual-role model for HMOX1, where moderate activation exerts a cytoprotective effect by scavenging ROS, while excessive activation increases labile Fe²⁺ levels, leading to ROS overload and ferroptosis. Further research is required to elucidate the precise role of HMOX1 in oleic acid-induced ferroptosis in MCECs. Collectively, our findings support the view that LD biogenesis serves as a protective mechanism by promoting the storage and limiting the release of polyunsaturated fatty acids, thereby reducing lipid peroxide formation, inhibiting ferroptosis, and maintaining cellular homeostasis in MCECs.

**Fig. 9:**
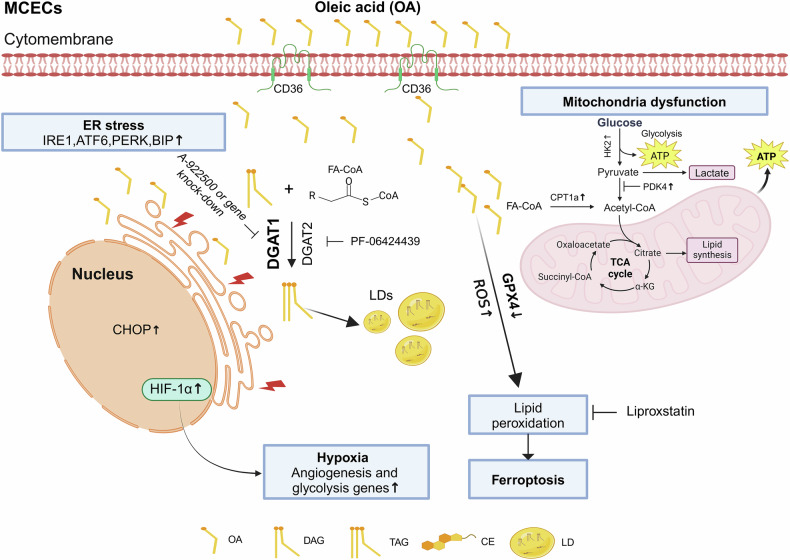
Diagram of DGAT1-mediated LDs formation in OA-induced endothelial dysfunction and ferroptosis in MCECs. OA oleic acid, LD lipid droplet, DAG diacylglycero, TAG triacylglycerol, CE cholesterol ester, FA-CoA fatty-acyl-CoA. Created in BioRender.com.

In summary, our work highlights the importance of LD biogenesis in mitigating oleic acid-induced lipotoxicity, EC homeostasis disruption, and ferroptosis. As depicted in Fig.9, excessive oleic acid accumulation in MCECs induces significant lipotoxic effects, leading to increased ER stress, mitochondrial damage, and hypoxia. Simultaneously, oleic acid overload increases lipid peroxidation, accompanied by downregulation of the key ferroptosis inhibitor GPX4. This downregulation amplifies lipid peroxide accumulation, thereby triggering ferroptosis. In another aspect, LD biogenesis is upregulated in response to oleic acid overload, primarily mediated by DGAT1 activity. LDs function as essential lipid-buffering and antioxidant organelles, sequestering free fatty acids, including polyunsaturated fatty acids, in triglycerides. This process reduces lipotoxicity and lipid peroxidation, preserving cellular homeostasis and preventing ferroptosis. These findings provide novel insights into the therapeutic potential of targeting DGAT1 activity to enhance LD biogenesis. Such strategies could offer a new avenue for preventing lipotoxicity-induced endothelial damage in vascular diseases, including coronary microvascular dysfunction associated with metabolic disorders.

## Materials and methods

### Antibodies and reagents

Primary antibodies: GPX4 (Abcam ab125066), PLIN2 (Proteintech 15294-1AP), β-actin (Cell Signaling Technology 3700S), SREBP1 (Abcam ab28481), SREBP2 (Santa Cruz Biotechnology sc-13552), CHOP (Cell Signaling Technologies 2895S), HIF1α (Cell Signaling Technology 36169S), IgG from mouse serum (Sigma I8765-5MG), IgG from rabbit serum (Sigma I5006-10MG).

Secondary antibodies for western blotting: IRDye® 800CW Donkey anti-Mouse IgG Secondary Antibody (Licor 926-32212), Anti-rabbit IgG, HRP-linked Antibody (Cell Signaling Technology 7074).

Secondary antibodies for immunofluorescence: Donkey anti-Rabbit IgG (H + L) Highly Cross-Adsorbed Secondary Antibody, Alexa Fluor 555™ (Thermo Fisher A-31572), Donkey anti-Rabbit IgG (H + L) Highly Cross-Adsorbed Secondary Antibody, Alexa Fluor 488™ (Thermo Fisher A-21206), Donkey anti-Mouse IgG (H + L) Highly Cross-Adsorbed Secondary Antibody, Alexa Fluor 555™ (Thermo Fisher A-31570).

Reagents: DMEM with sodium pyruvate and low glucose (Gibco 11885084), Oleic Acid (OA) (Cayman Chemical 90260), Liproxstatin-1 (LIP) (Cayman Chemical 17730), N-Acetyl cysteine (NAC) (Millipore Sigma A9165-25G), Z-VAD-FMK (Invivogen tlrl-vad), Z-DEVD-FMK (Cayman Chemical 14414), Ac-YVAD-cmk (Invivogen inh-yvad), Chloroquine (CQ) (Millipore Sigma C6628-25G), Bafilomycin A1 (BAF) (Millipore Sigma B1793-25UG), Spautin-1 (SP-1) (Millipore Sigma SML0440-5MG), (1S,3R)-RSL3 (RSL3) (Cayman Chemical 19288), PKUMDL-LC-101-D04 (PKUMDL) (Cayman Chemical 28966), A-922500 (DGAT1i) (Cayman Chemical 10012708), PF-06424439 (DGAT2i) (Cayman Chemical 17680), siLentFect™ Lipid Reagent for RNAi (Bio-Rad 1703362), BODIPY™ 493/503 (4,4-Difluoro-1,3,5,7,8-Pentamethyl-4-Bora-3a,4a-Diaza-*s*-Indacene) (Invitrogen D3922), DAPI (4’,6-diamidino-2’-phenylindole,dihydrochloride) (Thermo Scientific 62247), Cell Counting Assay (CCK-8) (ApexBio K1018), Seahorse XF Mito Stress Kit (Agilent 103015-100), Seahorse XF96 Cell Culture Microplate (Agilent 101085-004), Seahorse XF Calibrant pH 7.4 (Agilent 100840-000), Seahorse XFe96/XF Pro Extracellular Flux Assay Kit (Agilent Seahorse XF DMEM Medium pH 7.4 (Agilent 103575-100), Seahorse XF 100 mM Pyruvate Solution (Agilent 103578-100), Seahorse XF 200 mM Glutamine Solution (Agilent 103579-100), Seahorse XF 1.0 M Glucose Solution (Agilent 103577-100), MitoTracker Deep Red FM (Invitrogen M22426).

### Cell culture

The immortalized mouse cardiac endothelial cells (MCECs) line was purchased from Cedarlane (CLU510). MCECs were cultured in a low-glucose DMEM supplemented with 5% FBS, 1% penicillin/streptomycin, and 1 mM HEPES in a cell incubator containing 5% CO_2_ at 37 °C. Passages 43–50 of the MCECs were used in this study.

Human umbilical vein endothelial cells (HUVECs; Lonza, C2519AS) were maintained in Endothelial Cell Basal Medium-2 (EBM-2) supplemented with EGM-2 SingleQuots® (Lonza, CC-3162). For all treatments, HUVECs were serum-starved in EBM-2 containing 1% fetal bovine serum. HUVECs between passages 4 and 6 were used in this study.

### siRNA transfection

1 ×10^5^ MCECs were seeded in DMEM with 5% FBS for overnight. The following morning a transfection was performed when the cells were 50–70% confluent. After reaching 50–70% confluency, MCECs were transfected with 20 nM of si-*Ctrl* or si-*Dgat1* in DMEM with 5% FBS using SiLentFect LipidReagent (170-3361; Bio-Rad), according to the manufacturer’s instruction. After 24 h of transfection, the transfected MCECs were trypsinized down for further experiments or directly treated with OA in 1% FBS [75]. To be specific, 0.8 × 10^4^ transfected MCECs were seeded in 96-well plate for cell death study, 5 × 10^4^ transfected MCECs were seeded in 24-well plate with coverslips for BODIPY staining. DGAT1 mRNA level and OA-induced LD formation were used to confirm the silencing efficiency of si-*Dgat1*.

### Immunofluorescence staining

5 × 10^4^ MCECs were seeded in 5% FBS DMEM in 24-well plates with coverslips. After the desired treatment in 1% FBS containing DMEM, cells were washed with PBS and then fixed with 4% paraformaldehyde for 10 min at room temperature. Following fixation, cells were washed with PBS. Cells were then permeabilized and blocked in 5% BSA with 0.3% Triton X-100 in PBS at room temperature for 1 h. Cells were probed overnight with primary antibodies at 4 °C. An isotype-matched negative control antibody (mouse or rabbit IgG) was used at the same concentration as the primary antibody. Cells were washed in PBS containing 0.05% Tween-20 (PBS-T) and then incubated with secondary antibodies conjugated with Alexa Fluor 555, and/or Alexa Fluor 488, and the nuclear dye DAPI for 1 h at room temperature. Following incubation with the secondary antibody and DAPI, the cells were washed with PBS-T and mounted with Mowiol. For mitochondria staining, after treatment in DMEM with 1% FBS, cells were washed and the medium was replaced with serum-free DMEM containing 250 nM MitoTracker Deep Red FM (Invitrogen, M22426) and Hoechst 33342 (1:200 dilution). Staining proceeded for 30 min at 37 °C. Cells were then washed once with PBS, fixed in 4% paraformaldehyde for 10 min, washed again, and mounted in Mowiol prior to imaging. Images were taken as soon as possible using an Olympus IX73 imaging system or Leica Sted 8 imaging system, as described previously [76]. In Image-Pro Plus, DAPI images were used to generate nuclear AOI (areas of interest), which was then overlaid on the corresponding SREBP1, SREBP2, CHOP, or HIF1α channels to measure mean nuclear fluorescence intensity. Set positivity threshold of CHOP: *T* = *μ* + 2*σ*. *μ* = mean of OA group intensities, σ = standard deviation of OA group intensities. CHOP positive cells (%) = (number of CHOP-positive nuclei)/(total nuclei) × 100.

### BODIPY 493/503 staining

Lipid droplets were detected by the fluorescent dye BODIPY 493/503 (Invitrogen D3922). For microscopy, 5 × 10^4^ MCECs were seeded on a 24-well plate in DMEM with 5% FBS for overnight. The following day, the medium was changed to DMEM with 1% FBS for overnight. After the cells became fully confluent, they were treated with the desired concentration and time in DMEM with 1% FBS. After the desired treatment time, cells were washed with PBS and fixed with 4% paraformaldehyde for 10 min. After fixation, cells were incubated with BODIPY (40ug/ml) and DAPI in PBS-T for 30 min. Cells were washed with PBS-T after and then mounted with Mowiol. Images were taken as soon as possible using an Olympus IX73 imaging system. For microplate readings, 1–1.5 × 10^4^ MCECs were seeded in 5% FBS into a 96-well plate. After cells became fully confluent, they were treated with the desired concentration and time in DMEM with 1% FBS. Following treatment, the cells were incubated with 50 µL of a BODIPY/DAPI mix in a 96-well plate for 30 min. After washing with PBS-T, fluorescence is measured at 493 nm excitation and 503 nm emission using a microplate reader (BMG Labtech), as described previously [77].

### Cell Counting Assay (CCK-8)

0.8 × 10^4^ MCECs were seeded in DMEM with 5% FBS in a 96-well plate. The following day the cells were treated with indicated treatments in DMEM with 1% FBS. Plates were incubated for 2 h following the addition of 10 µL of CCK-8 (Apexbio K1018) solution according to the manufacturer’s instructions [22]. Absorbance (optical density, OD) was measured at 450 nm using a microplate reader (BMG Labtech).

### Western blotting

After the indicated treatment, MCECs were collected using 2× Laemmli sample buffer. The MCECs protein sample was boiled at 95 °C for 10 min and then sonicated in an ice water bath. 20–25 µg of total protein was separated by 12–15% SDS-PAGE. The proteins were transferred onto a PVDF membrane at 100 V room temperature for about 2 h or at 35 V overnight at 4 °C. After the transferred membrane was permeabilized with methanol and washed with TBS, the membrane was blocked with 5% BSA in TBS. The membrane was incubated with primary antibodies overnight at 4 °C. The membranes were then washed 3× with TBS-T and incubated with IRDye® 800CW or HRP secondary antibodies for 1 h at room temperature. Following 3× washes with TBS-T, the membranes were visualized and analyzed by the LI-COR Odyssey FC System, as described previously [78].

### Liperfluo staining of flow cytometry

12 × 10^4^ MCECs were seeded in DMEM with 5% FBS for overnight in a 12-well plate. MCECs were pre-treated with 5 µM DGAT1i for 30 min, then co-treated with or without 250 µM OA for 6 h. Following the treatment time, cells were stained with Liperfluo at 1:100 for 30 min. Then, the cells were trypsinized and fixed in 4% PFA. Cells were washed and stored in PBS to be used in flow cytometry. Cells were filtered prior to use for flow cytometry. Flow cytometry was run with the FITC channel on the Accuri C6 Plus Flow Cytometry System (BD Biosciences).

### Seahorse XF Mito Stress assay

Mitochondrial functions were measured by using the Seahorse XF Mito Stress Kit (Agilent 103015-100). 1.5 ×10^4^ MCECs were seeded into a seahorse 96-well plate (Agilent 101085-004) in DMEM with 5% FBS. The following afternoon, cells were changed to DMEM with 1% FBS or pretreated with 5 µM DGAT1i for overnight. The day prior to the assay, the sensor cartridge was hydrated in calibrant (Agilent 100840-000) for overnight. The morning after pretreatment, the cells are treated with different doses of OA with or without DGAT1i for 6H in DMEM with 1% FBS. The experiment was run according to the manufacturer’s instructions as a standard assay. The assay medium (Agilent 103575-100) was freshly prepared the day intended to run the assay and consisted of 1 mM pyruvate (Agilent 103578-100), 2 mM glutamine (Agilent 103579-100), and 5 mM glucose (Agilent 103577-100). The assay drugs: oligomycin, FCCP, and rotenone/antimycin A were prepared as described for stock concentrations. Final concentrations were as follows: oligomycin 2.5 µM, FCCP 2 µM, and rotenone/antimycin A 0.5 µM. One hundred and eighty microliters of assay medium with the desired concentrations of OA and DGAT1i were run in this assay. This assay was run on a Seahorse XFe96 Analyzer (Agilent Technologies). Mitochondria function parameters calculation: Non-mitochondrial respiration: Minimum rate measurement after Rotenone/antimycin A injection; Basal respiration: last rate measurement before first injection of oligomycin; Maximal respiration: (Maximal rate measurement after FCCP injection) – (non-mitochondrial respiration); Proton leak: (Minimum rate measurement after oligomycin injection) – (non-mitochondrial respiration); ATP production: (last rate measurement before first injection of oligomycin) – (Minimum rate measurement after oligomycin injection); Spare respiratory capacity: (Maximal respiration) – (Basal respiration); Spare respiratory capacity as a %: (Maximal respiration)/(Basal respiration) × 100; Coupling efficiency: (ATP production rate)/(Basal respiration rate) × 100.

### Quantitative real-time PCR

22 × 10^4^ MCECs were seeded in a 6-well plate in DMEM with 5% FBS. The following morning, cells were pretreated with 5 µM DGAT1i for 30 min, then cotreated with or without 250 µM OA in DMEM with 1% FBS. After 6 h of treatment, total RNA was extracted using the Aurum™ Total RNA Mini Kit (BioRad 732-6820). Once the RNA was extracted, the concentration was checked using a microplate reader (BMG Labtech). iScript Reverse Transcription Supermix was used to generate cDNA from the isolated total RNA for RT-qPCR (Bio-Rad, 1708841). The complete master mix was then incubated in a thermal cycler at 25 °C for 5 min to prime, 42 °C for 30 min to reverse transcribe, and 85 °C for 5 min to inactivate RT. The Real-Time PCR was performed using the iTaq Universal SYBR Green supermix (Bio-Rad, 1725121) on the Bio-Rad CFX Connect Real-Time System. An assay master mix was created containing 7.5 µL iTaq™ Universal SYBR^®^ Green Supermix, 5.5 µL nuclease-free water, and 1 µL primer. This 14 µL mix was added to each well of a 96-well PCR plate before 1 µL of cDNA was then added to each well. The plate was sealed and spun before a run was initiated on the CF Connect Real-Time System (Biorad). The run was as follows: denaturation at 95 °C for 2–5 s then an annealing/extension plate read for 15–30 s where both steps repeat for 40 cycles, followed by melt curve analysis at 65–95 °C in 0.5 °C increments, and lastly polymerase activation and DNA denaturation at 95 °C for 20-30 s. Primers used in the present study were listed in the supplementary Table 1. The cycle threshold values were converted to relative gene expression levels using the 2-ΔΔCt method. The data were normalized to internal control β-actin.

### Statistical analysis

Data are presented as Mean ± Standard Error of the Mean. Since none of our datasets met the normality assumption, the Kruskal–Wallis’s test was used, followed by Dunn’s multiple comparisons test. The Mann-Whitney test was applied for detecting significant differences between two groups. All statistical analyses were conducted using GraphPad Prism 6.0 (GraphPad Software, USA), with *P* < 0.05 considered statistically significant. The sample size of n = 4 per group was based on prior studies and pilot data showing low variability in our system. This number is commonly used in cell-based experiments and provides sufficient power to detect moderate to large effects. Experiments were also independently repeated at least 3 times to ensure reproducibility. Samples were excluded if they exhibited contamination, technical issues, or if data points were identified as outliers based on pre-defined statistical thresholds or measurement errors. All inclusion and exclusion criteria were pre-established to ensure consistency and maintain high data quality across experiments. The investigator was blinded to the group allocation during both the conduct of the experiment and outcome assessment to minimize bias and ensure objective data interpretation.

## Supplementary information

Supplementary Figures Legend

Supplementary Figure 1.

Supplementary Figure 2.

Original Data-Supplementary Figure 3

Supplementary table 1

## Data Availability

All data and supporting materials have been provided with the published article and its online supplementary files.
