# Supplementary material for: Defective lipid droplet biogenesis exacerbates oleic acid-induced cellular homeostasis disruption and ferroptosis in mouse cardiac endothelial cells
Source: Cell Death Discov. 2025 Aug 9;11:374. doi: 10.1038/s41420-025-02669-5 (PMC12335489; doi:10.1038/s41420-025-02669-5)
Supplement: Supplementary file 1 — Supplementary Figures Legend [file 41420_2025_2669_MOESM1_ESM.docx]

**Supplemental Information**

**Defective Lipid Droplet Biogenesis Exacerbates Oleic Acid-Induced Cellular Homeostasis Disruption and Ferroptosis in Mouse Cardiac Endothelial Cells**

Yun-Ting Wang ^1*^, Alexandra K Moura ^1*^, Rui Zuo ^1^, Zhengchao Wang ^1,3^, Kiana Roudbari ^1^, Jenny Z. Hu ^1^, Mi Wang ^1,4^, Pin-Lan Li^2^, Yang Zhang ^1#^, Xiang Li ^1#^

1, Department of Pharmacological and Pharmaceutical Sciences, College of Pharmacy, University of Houston, Houston, USA

2, Department of Pharmacology and Toxicology, Virginia Commonwealth University, School of Medicine, Richmond, VA, USA

3, Provincial Key Laboratory for Developmental Biology and Neurosciences, College of Life Sciences, Fujian Normal University, Fuzhou, China

4, Department of Gastroenterology and Hepatology, Tongji Hospital, Tongji Medical College, Huazhong University of Science and Technology, Wuhan, Hubei, China

Running title: LD in oleic acid-induced mitochondrial dysfunction and ferroptosis

* Contributed equally

**#** Corresponding authors

**Correspondence to**: Xiang Li, M.D., Ph.D., Department of Pharmacological & Pharmaceutical Sciences, College of Pharmacy, University of Houston, Houston, TX 77204-5056, Tel: 7137437710, Fax: 7137431259, Email: [xli61@central.uh.edu](mailto:xli61@central.uh.edu); or Yang Zhang, Ph.D., Department of Pharmacological & Pharmaceutical Sciences, College of Pharmacy, University of Houston, Email: [yzhan219@central.uh.edu](mailto:yzhan219@central.uh.edu)

**Supplementary Figures Legend**

**Supplementary Figure 1.** MCECs were seeded overnight, and upon reaching 70% confluence, were pre-treated with indicated inhibitors for 1 hour, followed by treatment with 300 µM OA for overnight. Effect of pan-caspase inhibitor (Z-VAD-FMK) (**A**), caspase-3 inhibitor (Z-DEVD-FMK) (**B**), caspase-1 inhibitor (AC-YVAD-CMK) (**C**), lysosome inhibitor chloroquine (CQ) (**D**) and bafilomycin (BAF) (**E**), and autophagy inhibitor spautin-1 (SP-1) (**F**) on OA-induced cell death. All datasets were analyzed by the Kruskal–Wallis test with Dunn’s multiple-comparisons post hoc test. P < 0.05 vs. corresponding CTRL lacking OA and additional treatments. n =4. OD, optical density.

**Supplementary Figure 2. DGAT1-mediated LD biogenesis protects against OA-induced ferroptosis in cultured HUVECs.** HUVECs were seeded overnight, and upon reaching 70% confluence, were pre-treated with or without indicated inhibitors for 30 minutes, followed by treatment with indicated doses OA for specified times. **A and B**. Bodipy staining and plate-read fluorescence intensity quantification show DGAT1 inhibition blocked 100µM OA-induced LD formation in HUVECs. **C**. 250 µM OA significantly induced cell death in HUVECs after overnight treatment. **D**. DGAT1 inhibition enhanced OA-induced cell death, which could be rescued by LIP. **E.** pan-caspase inhibitor (50µM Z-VAD-FMK), caspase-3 inhibitor (20µM Z-DEVD-FMK), caspase-1 inhibitor (30ug/ml AC-YVAD-CMK), lysosome inhibitor chloroquine (5µM CQ) and bafilomycin (10nM BAF), and autophagy inhibitor spautin-1 (10µM SP-1) has no effect on OA and DGAT1i-induced cell death. Data are shown as fold‐changes relative to control without OA treatment (CTRL = 1). Images in Fig. A acquired using a 40× objective and a standard 10× eyepiece (effective 400× magnification). Scale bar=20 µm. All datasets were analyzed by the Kruskal–Wallis test with Dunn’s multiple-comparisons post hoc test. In panel B, * P < 0.05 vs. corresponding CTRL without OA (0µM OA). In panel C, * P < 0.05 vs. corresponding CTRL with 100 µM OA; # P < 0.05 vs. 100 µM OA+DGAT1i. n = 4. OD, optical density. RFU, relative fluorescence unit.

**Supplementary Figure 3. Full and uncropped immunoblot corresponding to Fig. 1H.**
