# Supplementary figures and images for: Defective lipid droplet biogenesis exacerbates oleic acid-induced cellular homeostasis disruption and ferroptosis in mouse cardiac endothelial cells
Source: Cell Death Discov. 2025 Aug 9;11:374. doi: 10.1038/s41420-025-02669-5 (PMC12335489; doi:10.1038/s41420-025-02669-5)

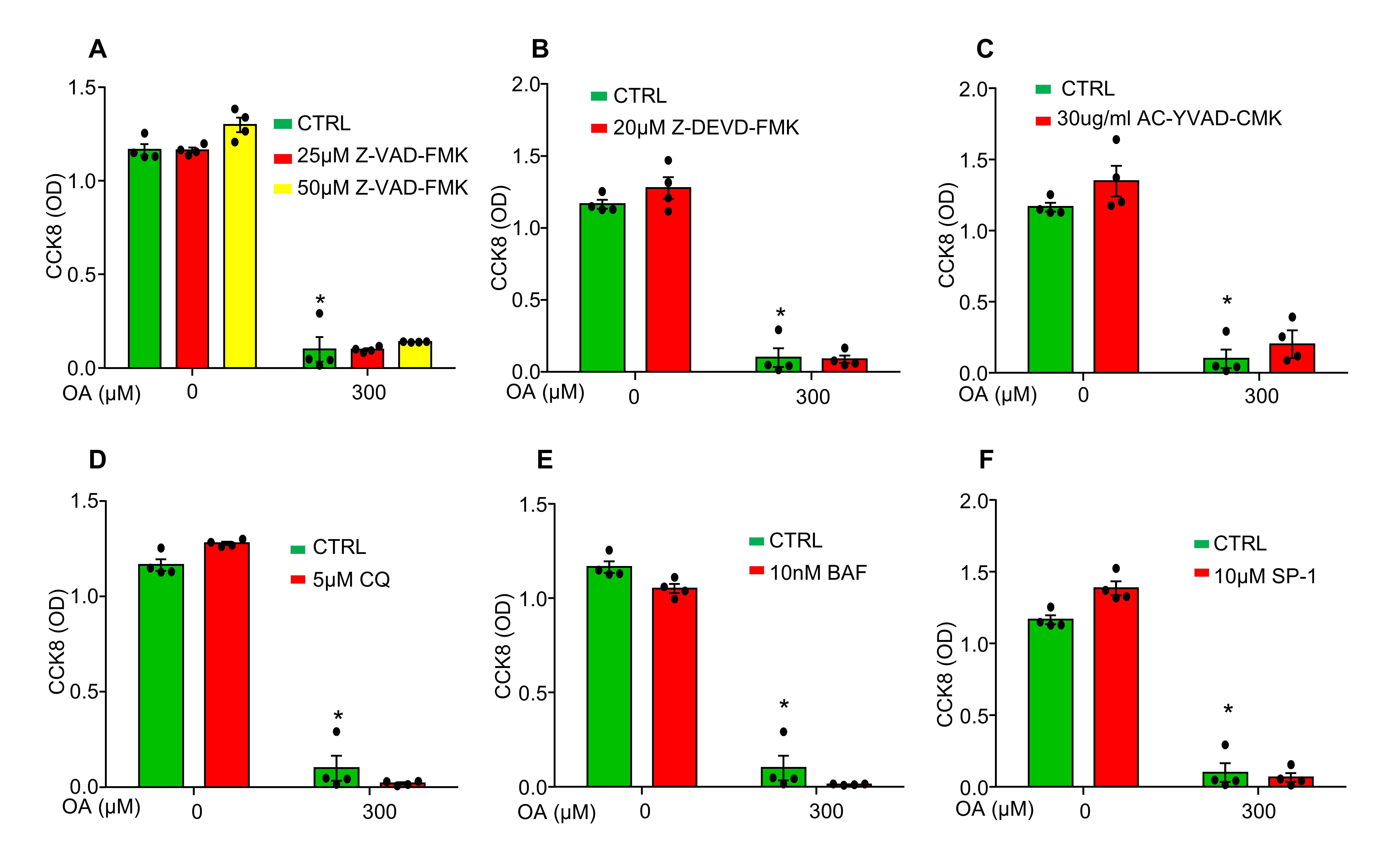

Supplement: Supplementary file 2 — Supplementary Figure 1. [file 41420_2025_2669_MOESM2_ESM.jpg]

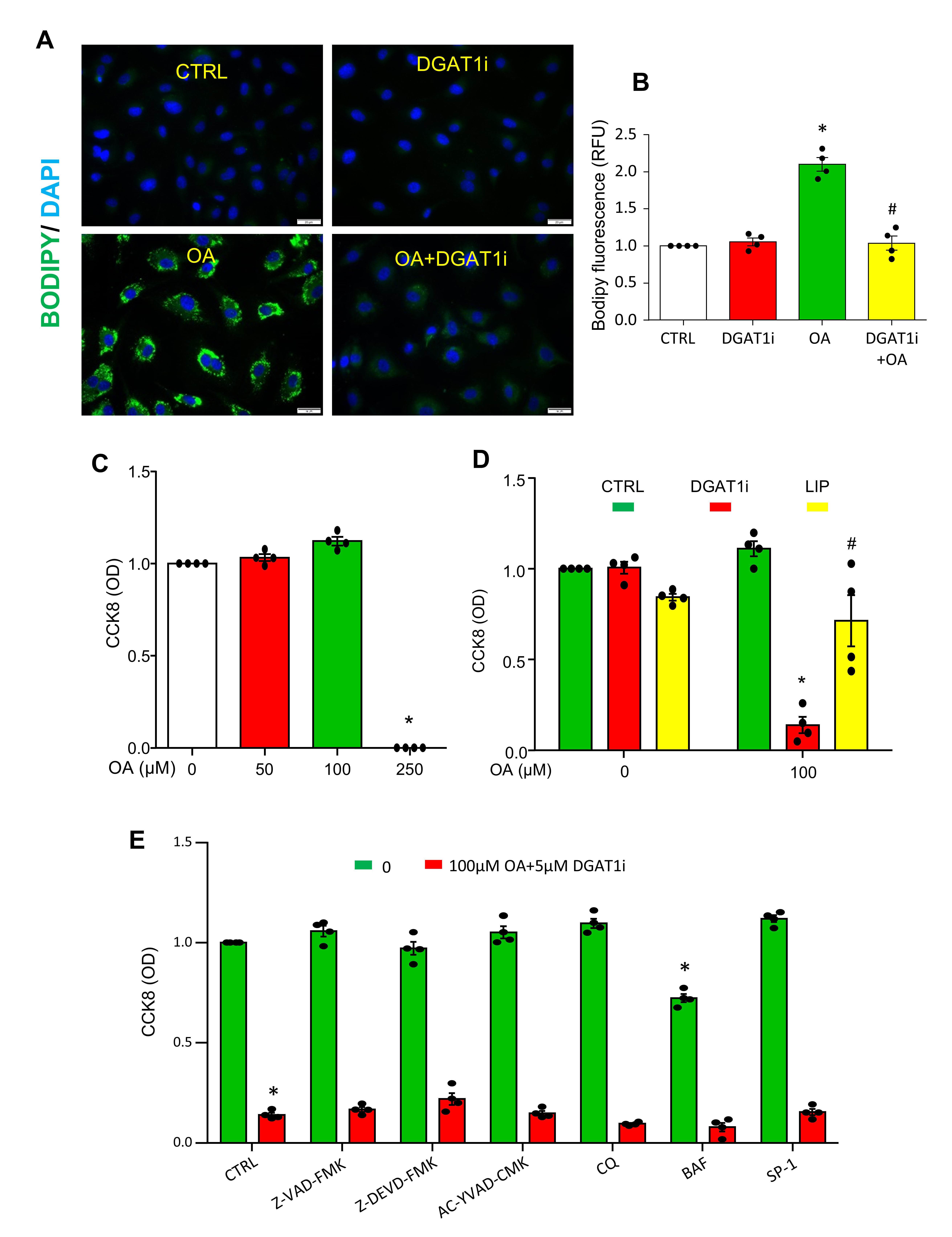

Supplement: Supplementary file 3 — Supplementary Figure 2. [file 41420_2025_2669_MOESM3_ESM.jpg]
