# Supplementary material for: Defective lipid droplet biogenesis exacerbates oleic acid-induced cellular homeostasis disruption and ferroptosis in mouse cardiac endothelial cells
Source: Cell Death Discov. 2025 Aug 9;11:374. doi: 10.1038/s41420-025-02669-5 (PMC12335489; doi:10.1038/s41420-025-02669-5)
Supplement: Supplementary file 5 — Supplementary table 1 [file 41420_2025_2669_MOESM5_ESM.docx]

Supplementary table 1: Primer list.

| Primer (m) | Forward (5’-3’) | Reverse (5’-3’) |
| --- | --- | --- |
| *Plin2* | CGTCTGTCTGGACCGAATAAAG | CACACGCCTTGAGAGAAACA |
| *Srebf1* | CAGACTCACTGCTGCTGACA | GATGGTCCCTCCACTCACCA |
| *Fasn* | TGCACCTCACAGGCATCAAT | GTCCCACTTGATGTGAGGGG |
| *Acaca* | CGCCAGCCTGAGTTCTTTT | TTTGGCCAACGGAGATGGTT |
| *Scd1* | GGAGACGGGAGTCACAAGAG | TGCATCATTAACACCCCGAT |
| *Fads1* | CAACATCAGCGACTTCAGCC | CCACAAAAGGATCCGTGGCA |
| *Atgl* | ACCTTCGCAATCTCTACCGC | TGGGTTGGTTCAGTAGGCCA |
| *Lipa* | GTAGGTGTAGGCACCAGGTT | CATCTTCCGGGAGTGGTCCT |
| *Ppard* | AAACCCACGGTAAAGGCAGT | CTGTTCCATGACTGACCCCC |
| *Pdk4* | CAGCTGCTGGACTTTGGTTCA | TTCAGGATATTGGCCAGGCG |
| *Cpt1a* | GACTCCGCTCGCTCATTCC | ACCAGTGATGATGCCATTCTTG |
| *Cpt2* | GTATCTGCAGCACAGCATCG | TTTAGGGATAGGCAGCCTGG |
| *Slc25a20* | CCTGGACACGGTCAAGGTCC | CGGAAACAGTCCAAGGTCCC |
| *Gpx4* | GCCGTCTGAGCCGCTTACTTA | CCTTGGCTGAGAATTCGTGC |
| *Fth1* | GGGCTGAATGCAATGGAGTG | TCAATGAAGTCACATAAGTGGGG |
| *Lpcat3* | CCCTTCATTGCTGGAAGTTGC | GAGCAGGTATGGTGCTGTTTG |
| *Acsl4* | GCACCTTCGACTCAGATCACA | AGCCAGCAATAAAGTACACAGAT |
| *Slc3a2* | TTTAGGGGAGTGCGACGCTA | CGCGAACCAAAATCTCCAGC |
| Slc7a11 | AAATACGGAGCCTTCCACGA | CTCCAGGGGCAGTCAGTTAG |
| *Hspa5* | TCGATACTGGCCGAGACAAC | CGACGGTTCTGGTCTCACAC |
| *Trib3* | AGCACTTTAGCAGCGGAAGA | AGGTGTAGCTCGCATCTTGT |
| *Chac1* | AGTGTGGAAGCCGGACTTTG | CACTCGGCCAGGCATCTTGT |
| *Ddit3* | CCTGAGGAGAGAGTGTTCCAG | GACACCGTCTCCAAGGTGAA |
| *Pp1r15a* | CTCTAAAAGCTCGGAAGGTACAC | GGCTTCGATCTCGTGCAAAC |
| *Atf3* | GTCACCAAGTCTGAGGCGG | GTTTCGACACTTGGCAGCAG |
| *Eif2s1* | TAAGCATGCAGTCTCAGACCC | CTTGTGGGGTCAAACGCCTA |
| *Eif2ak3* | CCGCAAGAAGGACCCTATCC | CAGACTCCTTCCGCTGCCTG |
| *Atf6* | GGGAGAGGTGTCTGTTTCGG | AAACAACGTCGACTCCCAGT |
| *Ern1* | TGGCTTCTCATAGGACACCAT | TTCTCGATGTTTGGGCAGGT |
| *Hif1α* | TGGACTTGTCTCTTTCTCCGC | CGACGTTCAGAACTCATCCT |
| *Hif1β* | TATTAAGCGACGGTCAGGGC | CTGCTCATCATCCGACCTGG |
| *Epas1* | CTGAGGAAGGAGAAATCCCGT | TGTGTCCGAAGGAAGCTGATG |
| *Siah2* | ACCAATGCCGCCAGAAGTTA | CAGCCCGTGGTAGCATACTTA |
| *Egln1* | AGGCTATGTCCGTCACGTTG | TACCTCCACTTACCTTGGCG |
| *Egln2* | GGAGGAAAAAGCTCGCCAC | CAGCAGTGTCTTCATCCCCC |
| *Egln3* | AAGGAGCGGTCCAAGGCAAT | ATACAGCGGCCATCACCATT |
| *Hilpda* | TTCCTTACTCCTGCACGACCT | ATGATGCCCAGCACATAGAGG |
| *Vegfa* | CTTGTTCAGAGCGGAGAAAGC | ACATCTGCAAGTACGTTCGTT |
| *Hmox1* | CAGAGCCGTCTCGAGCATAG | CAAATCCTGGGGCATGCTGT |
| *Hk2* | CTGTTTCTGGAAACTTGAGGCCC | AGAGATACTGGTCAACCTTCTGC |
| *Angptl4* | CACCCACTTACACAGGCCG | GAAGTCCACAGAGCCGTTCA |
| *Fgf21* | CCTTGAAGCCAGGGGTCATT | GGATCAAAGTGAGGCGATCC |
